# Ultrahigh-Throughput Virtual Screening Strategies against PPI Targets: A Case Study of STAT Inhibitors

**DOI:** 10.1021/acs.jcim.5c00907

**Published:** 2025-07-04

**Authors:** Tibor Viktor Szalai, Nikolett Péczka, Levente Sipos-Szabó, László Petri, Dávid Bajusz, György M. Keserű

**Affiliations:** 1 Medicinal Chemistry Research Group and Drug Innovation Centre, 579839 HUN-REN Research Centre for Natural Sciences, Magyar tudósok krt. 2, 1117 Budapest, Hungary; 2 Department of Inorganic and Analytical Chemistry, Faculty of Chemical Technology and Biotechnology, Budapest University of Technology and Economics, Műegyetem rkp. 3, H-1111 Budapest, Hungary; 3 Department of Organic Chemistry and Technology, Faculty of Chemical Technology and Biotechnology, Budapest University of Technology and Economics, Műegyetem rkp. 3, H-1111 Budapest, Hungary

## Abstract

In recent years, virtual screening of ultralarge (10^8+^) libraries of synthetically accessible compounds (uHTVS) became a popular approach in hit identification. With AI-assisted virtual screening workflows, such as Deep Docking, these protocols might be feasible even without supercomputers. Yet, these methodologies have their own conceptual limitations, including the fact that physics-based docking is replaced by a cheaper deep learning (DL) step for the vast majority of compounds. In turn, the performance of this DL step will highly depend on the performance of the underlying docking model that is used to evaluate parts of the whole data set to train the DL architecture itself. Here, we evaluated the performance of the popular Deep Docking workflow on compound libraries of different sizes, against benchmark cases of classic brute-force docking approaches conducted on smaller libraries. We were especially interested in more difficult, protein–protein interaction-type oncotargets where the reliability of the underlying docking model is harder to assess. Specifically, our virtual screens have resulted in several new inhibitors of two oncogenic transcription factors, STAT3 and STAT5b. For STAT5b, in particular, we disclose the first application of virtual screening against its N-terminal domain, whose importance was recognized more recently. While the AI-based uHTVS is computationally more demanding, it can achieve exceptionally good hit rates (50.0% for STAT3). Deep Docking can also work well with a compound library containing only several million (instead of several billion) compounds, achieving a 42.9% hit rate against the SH2 domain of STAT5b, while presenting a highly economic workflow with just under 120,000 compounds actually docked.

## Graphical abstract

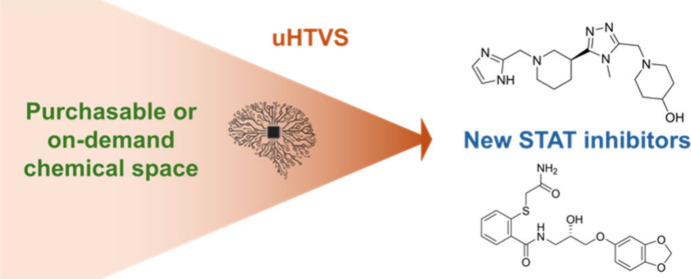

## Introduction

Searching “synthetically accessible” or “make-on-demand” ultralarge (10^8+^ compounds) chemical databases^1,2^ provides a unique opportunity to sample the corresponding chemical space effectively. Ultrahigh-throughput virtual screening (uHTVS) methods^3^ designed for these applications leverage the much increased computational demand with a logical layer, such as a deep learning model^4–7^ or a bottom-up (e.g., fragment- or synthon-based) generative approach.^8–10^ Contemporary, large chemical libraries of compound vendors and aggregators like Enamine,^11^ Mcule,^12^ and eMolecules^13^ now routinely offer billions (or even trillions) of synthesizable virtual compounds. As the “brute-force” evaluation (docking every compound in the library) of these libraries is seriously demanding (or, without the proper infrastructure, completely unfeasible), efforts were made to reduce the computational cost of uHTVS without losing a significant amount of true hits. These efforts include enhancing the speed of docking by using GPUs and input-output optimization^14–16^ or using integrated workflows that reduce the number of actually docked compounds (and thereby, the computational cost) by using iterative machine learning (ML) to generate a deep neural network model^4–7^ or a synthon-based concept for combinatorial library generation.^8–10^ The uHTVS method might increase the chance of identifying new, potent inhibitors. This is particularly advantageous for targeting protein–protein interactions (PPIs), which is widely considered to be more challenging due to the lack of a single, deep, and well-defined binding cavity. At the same time, the performance of AI-based uHTVS methods is highly dependent on the performance of the underlying docking model (that is used to train the deep learning model itself), which is considered to be worse for PPI targets for the very same reason. Therefore, we intended to test the performance of contemporary uHTVS methods, particularly Deep Docking, on STAT3 and STAT5b, which are a pair of relevant PPI-type oncotargets. A traditional workflow of "brute-force" docking with smaller compound libraries is performed as a benchmark, and finally, the most cost-effective setup is applied to discover new inhibitors of the N-terminal domain of STAT5b, which was recently established as a promising pharmaceutical target.^17^

Signal transducer and activator of transcription (STAT) proteins are a family of transcription factors with key roles in cytokine signaling, growth factor stimulation, and DNA transcription activation.^18^ There are seven identified members of the STAT protein family (STAT1, STAT2, STAT3, STAT4, STAT5a, STAT5b, and STAT6), each having a unique role in cytokine signaling. When the physiological significance of each STAT protein in ‘knockout’ mice *in vivo* was investigated,^19^ the absence of STAT1 or STAT2 proteins caused a lack of immune response to interferons, making the living organism more susceptible to infections.^19–22^ Absence of STAT3 could not be investigated in mice, as it caused embryonic lethality;^19,23^ however, a different study^24^ discussed the role of STAT3 in the migration of keratinocyte cell migration in skin,^25^ in the activation of T cells by interleukin-6,^26^ in the signaling of macrophages,^27^ and in the apoptosis of mammary gland cells.^28^ STAT4 and STAT6 proteins have roles in T lymphocyte development by activation through some types of cytokines (particularly interleukin-4, interleukin-12, and interleukin-13), and the lack of these proteins resulted in the living organism being unresponsive to these cytokines.^19^ Absence of STAT5a resulted in impaired mammary gland development and lactogenesis^29^ and an impairment in peripheral T lymphocyte proliferation^30^ while absence of STAT5b resulted in a major loss of multiple, sexual dimorphisms.^31^

Although STAT proteins have different roles, they have a highly conserved, modular structure with six domains. The N-terminal domain (NTD) is used for higher-order homo- and heterodimerization, the coiled-coil domain (CCD) interacts with other proteins which participate in nuclear import and export, the DNA binding domain (DBD) is used to identify and bind to the target gene palindrome sequence, the linker domain (LD) participates in the phosphorylation of the protein, the Src Homology 2 (SH2) domain is used to identify phosphotyrosine sites and regulates the protein–protein interactions (PPIs), stabilizing STAT homodimer formation through phosphotyrosine-SH2 interactions, and last the transcription activation domain (TAD) found at the C-terminal end contains the tyrosine and serine phosphorylation sites required for gene transcription activation.^32–34^ Out of the six domains, the most commonly targeted is the SH2 domain for its well-defined phosphotyrosine-binding (pY) site with a conserved arginine residue and its role in downstream signaling.^35–37^

STAT proteins, mainly STAT1, STAT3, and STAT5b, exhibit oncological properties.^18,34,38,39^ STAT1 has a role as an inhibitor of cell proliferation and assists apoptosis, acting as a tumor suppressor, and its absence caused mice to be more susceptible to carcinogen-induced tumors.^40–45^ STAT3 and STAT5b proteins are necessary for cancer cell formation and cell survival, and their mutations and overexpression can initiate cancer-related processes.^18^ STAT3 is associated with many types of cancer, including leukemias, melanoma, and prostate cancer,^17,18,34,39^ while STAT5b protein is associated with breast cancer, colorectal cancer, lung cancer, prostate cancer, and leukemias.^18,46^ STAT3 and STAT5b are key oncological targets, as their inhibition causes cancer-derived cells to undergo growth arrest or apoptosis, while healthy cells are mainly unaffected.^18,47^

Identifying potent small-molecule STAT inhibitors is challenging due to its large, solvent-exposed PPI interface.^48^ Virtual screening is a promising method to identify potent STAT inhibitors, as it is a cost-effective alternative to experimental high-throughput screening, and it can achieve much higher hit rates.^49,50^ To assist the identification of potent inhibitors, chemical databases targeting a specific domain have been compiled, like the SH2 Domain Targeted Library of OTAVAchemicals, which contains drug-like compounds with a predicted affinity to SH2 domains based on generic pharmacophore patterns.^51^ Due to the increased 3D-likeness and complexity of natural products, screening such libraries represents another knowledge-based option to identify hits against PPI targets.^52,53^

In this study, we have examined the performance of the Deep Docking workflow against the STAT3 SH2 domain and benchmarked it against a virtual screen of a diversity-picked subset of the Mcule-in-stock data set (with its size matching the number of compounds docked in the Deep Docking workflow itself). Additionally, we have performed ‘traditional’, brute-force virtual screens against two smaller libraries (OTAVAchemicals^51^ SH2 Domain Targeted Library and a natural product library) as an alternative, ‘knowledge-based’ approach. The AI-based tool Deep Docking^4^ was used to recover virtual hits from the Enamine REAL library^11^ and the Mcule-in-stock library.^12^ The aims of this study were to examine whether uHTVS with an AI-based tool can reach significantly better hit rates than a ‘knowledge-based’ approach, and also to examine if AI-based tools can perform well even after training the deep learning model with less than 100,000 (instead of millions) compounds (economic screening workflow). Against STAT3-SH2, we have found Deep Docking to be capable of reaching exceptional hit rates, as high as 50.0%. Additionally, it can also perform well with the smaller Mcule-in-stock library containing ‘only’ millions (instead of billions) of compounds. Finally, the performance of this economic screening workflow was confirmed in prospective studies, identifying ligands of STAT5b SH2 and N-terminal domains. In addition to identifying new STAT inhibitors, this study highlights the effectiveness of uHTVS with an AI-based tool and provides a benchmark study for using AI-based tools with a smaller compound library.

## Methods

### Data Sets for the ‘Knowledge-Based’ Approach

Two data sets were examined for the ‘knowledge-based’ approach: the first containing 1,807 compounds specifically collected by generating and clustering pharmacophore models interacting with the SH2 domain (OtavaSH2 data set),^51^ the second containing 193,757 naturally occurring, or natural product-like compounds (NP data set) compiled from several vendors: LifeChemicals,^54^ ChemBridge,^55^ Asinex,^56^ and ChemDiv.^57^ Pan-assay interference compounds (PAINS compounds) were filtered out from these data sets in advance.^58^

### Data Sets for the AI-Based Approach

Two data sets were examined for the AI-based approach. The Enamine REAL data set contains 5.51 billion compounds, each complying with Lipinski’s rule of five^59^ and the Veber criteria^60^ and each compound being synthetically accessible. The Mcule-in-stock data set contains 5.59 million compounds, each being in stock and purchasable from Mcule. The Benchmark data set contained 117,500 chemically diverse compounds, selected from the Mcule-in-stock data set with the RDKit^61^ Diversity Picker node^62^ in KNIME.^63^ Just as for the data sets used in the ‘knowledge-based’ approach, the only filtering made in advance was to filter out PAINS compounds.

### Docking and Virtual Screening

#### STAT3 SH2 Domain

The appropriate X-ray structure for docking was selected by carrying out a retrospective virtual screening with a small data set containing 69 known actives for STAT3 from ChEMBL^64^ and 959 decoy molecules generated with the DUD-E database^65^ and evaluating the performance metrics AUC (Area Under the ROC Curve) and 1%, 2%, and 5% EF (Enrichment Factor) values based on the docking scores for each structure and docking setting. Preparation of the data set was carried out with LigPrep^66^ at a pH range of 7.4 ± 1.0, and the docking was performed with Glide^67,68^ single precision (SP) mode using different H-bond constraint settings inside the SH2 domain. Based on the performance metrics, the X-ray structure with the PDB ID 6QHD^69^ was used with an H-bond constraint (with Glide) with the R609 residue for all virtual screening runs against STAT3 SH2 domain. R609 has been shown earlier to function as a key anchoring residue of the SH2 domain in STAT3.^70^ Performance metrics such as AUC and enrichment factor values (EF) for 6QHD are included in Table 1, while the ROC curve is included in Supporting Information Figure S1. The training set of known actives and decoys are included in Supplementary Data.

**Table 1:** Performance Metrics for 6QHD

Performance metric	Value
AUC	0.887
EF(1%)	5.005
EF(2%)	6.674
EF(5%)	7.341

Ligand preparation for the OtavaSH2, NP, and Mcule-in-stock data sets were also carried out with LigPrep at a pH of 7.4 ± 1.0, and the docking calculations were done using Glide in SP mode. In the case of Enamine REAL, ligand preparation was carried out with the open-source programs Dimorphite-DL^71^ and RDKit,^72^ grid generation was carried out with AutoDockTools and AutoGrid,^73^ and the docking was carried out using AutoDockGPU.^74^ Despite its generally worse performance to Glide, AutoDockGPU was chosen in this case as an open-source alternative to significantly speed up the docking steps for the much larger Enamine REAL data set, utilizing GPU acceleration on an HPC cluster. As AutoGrid does not have the capacity to set H-bond constraints, docking with AutoDockGPU was performed without constraints.

Additionally, Deep Docking was also used with the data sets used for the AI-based approach, coupled with the appropriate docking algorithm (AutoDockGPU for Enamine REAL, Glide for Mcule-in-stock). Each Deep Docking workflow consisted of 11 iteration cycles, each with 7,500 compounds to be docked (in the first iteration, 3 × 7,500 compounds are docked for training, test, and validation sets) for the Mcule-in-stock data set, and each with 1,000,000 compounds to be docked (3 × 1,000,000 in the first iteration) for the Enamine REAL data set. For all Deep Docking runs, the number of hyperparameters was set to 12, the training time was set to a maximum of four hours, and the recall value was set to 0.9, i.e. the default choices described in the original paper.^4^ Deep Docking workflows were carried out using an in-house script that was published on our Github page (https://github.com/keserulab/uHTVS_toolkit) as part of our recent work.^75^

#### STAT5b SH2 Domain

The X-ray structure with the PDB ID 6MBW^76^ was used for docking purposes as it was the only wild-type STAT5b X-ray structure available at the time. The Mcule-in-stock data set was prepared as described before. We note here that the Enamine REAL data set was not used for compound selection against the STAT5b SH2 domain as we deemed the Mcule-in-stock data set sufficient for the AI-based approach, based on our experience with the STAT3 SH2 domain.

For virtual screening, an H-bond constraint with the R618 residue was set as the analogue for the R609 residue in the STAT3 SH2 domain. To evaluate the goodness of the used receptor grid, a retrospective virtual screening using a data set containing 28 known actives for STAT5b found in the literature^77–82^ and 1650 decoy molecules generated with DUD-E^65^ was used, and the performance metrics AUC and EF values belonging to the best 1%, 2%, and 5% of compounds based on docking score were evaluated. Virtual screening settings for the AI-based approach were the same as described for the STAT3 SH2 domain. Performance metrics such as AUC and EF values for 6MBW are included in Table 2, while the ROC curve is included in Supporting Information Figure S2. The training set of known actives and decoys are included in Supplementary Data.

**Table 2:** Performance Metrics for the 6MBW

Performance metric	Value
AUC	0.850
EF(1%)	25.98
EF(2%)	12.60
EF(5%)	9.302

#### STAT5b NTD

As described earlier, the STAT5b NTD is a novel, promising target for STAT5b inhibition, as it can inhibit the higher-order oligomerization processes of the STAT5b protein. As the information about this target is scarce, a ‘knowledge-based’ approach was not carried out for this target, only an AI-based approach utilizing the experiences gathered during the SH2 domain case studies, i.e., using the Mcule-in-stock data set and Glide SP as the docking algorithm. In lack of a resolved experimental structure for the N-terminal domain at the time, the protein structure used for the virtual screening tasks was a homology model generated using the Prime protein modeling program based on the STAT3 N-terminal domain structure (PDB: 4ZIA).^83–85^

Preparing the Mcule-in-stock data set was carried out the same way as described earlier. A docking grid was generated to include the handshake dimer interface, and no constraint was set. For the Deep Docking workflow, 22,500 compounds were docked in each iteration (3 × 22,500 in the first iteration), with the other Deep Docking settings kept as described earlier.

#### Overall Workflow

Figure 1 shows an overview of the used workflows, targets, and data sets in the current work. After the performance evaluation of the used structures for STAT3 and STAT5b (described earlier), first the virtual screening runs against STAT3 were carried out, and they were evaluated based on hit rates and the used resources. The AI-based workflow with the Mcule-in-stock data set was found as a good balance between performance and resource usage, and it was shown to convincingly outperform the benchmark VS workflow that consisted of docking the same number of diversity-picked compounds from the same data set (i.e., without AI augmentation). Thus, the Deep Docking workflow was applied prospectively for the virtual screening runs first against the STAT5b SH2 domain and then against the novel target STAT5b NTD.

**Figure 1:**
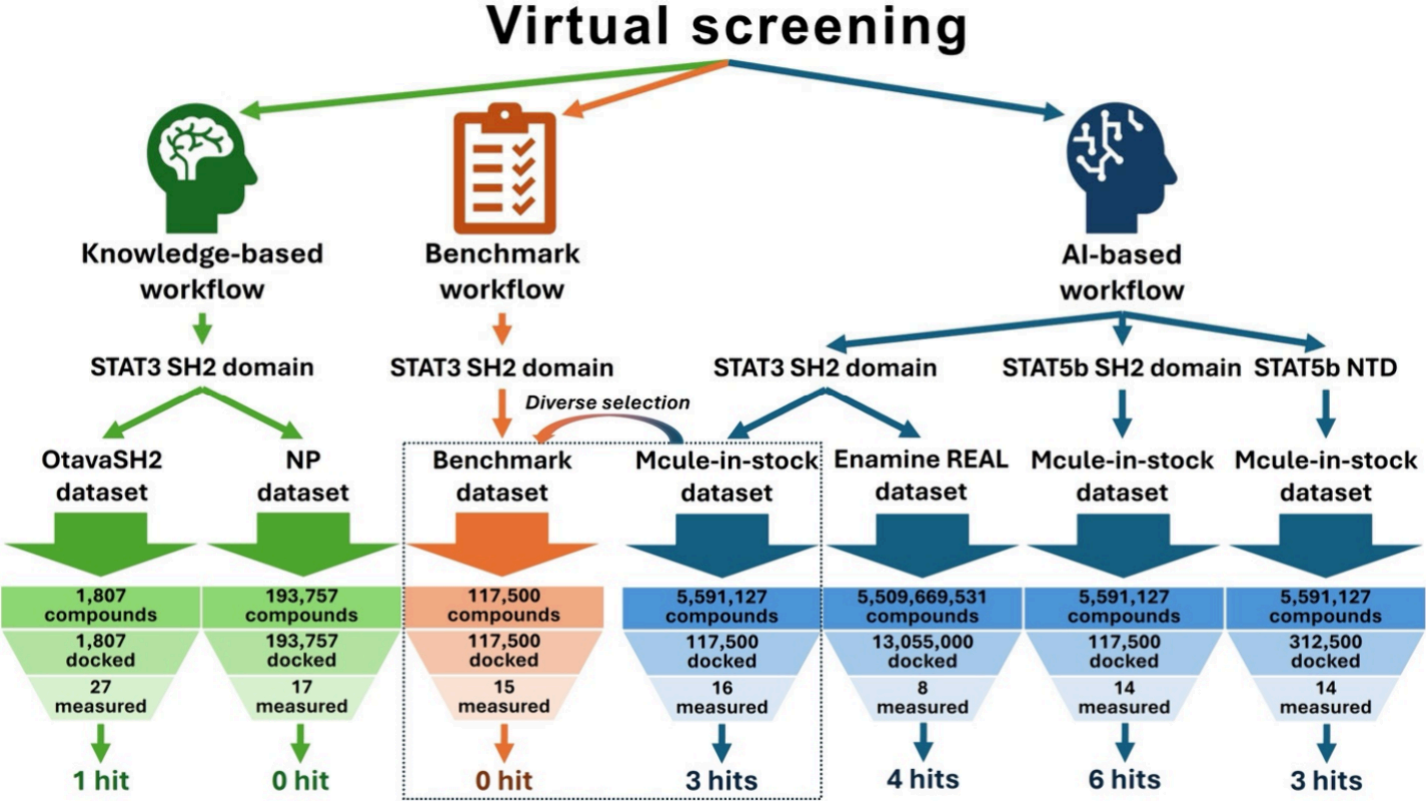
Overview of the applied ‘knowledge-based’ and AI-based virtual screening workflows against the appropriate targets. Green and blue colorings highlight the ‘knowledge-based’ and AI-based approaches, respectively. (The orange data set represents a benchmark against the AI-based STAT3/Mcule-in-stock virtual screen, where the Deep Docking workflow is replaced by a simple diversity selection.).

#### Compound Selection

Compound selection was performed using the output from each virtual screening workflow. In the case of the ‘knowledge-based’ approach, this corresponds to the output acquired from the docking of the whole data sets. For the AI-based approach, first the top 20,000 (Mcule-in-stock) or 55,000 (Enamine REAL) predicted virtual hits based on their Virtual-Hit Likeness value (VHL value or also termed as p-value within the workflow) after the last iteration of the Deep Docking workflow were exported, then they were docked with Glide SP with an appropriate H-bond constraint, and the output from these dockings was used. Glide SP docking with an appropriate H-bond constraint was used for compound selection also for the Enamine REAL data set, based on our recent findings regarding the better pose prediction performance of Glide vs AutoDockGPU.^75^

Compound selection was started by ranking the compounds based on (i) docking scores and (ii) docking score/heavy atom count (DS/HA) values. After the ranking, the binding modes were visually inspected until the desired number of compounds was selected, starting with the best (most negative) docking score or DS/HA values and working our way up. A total of 30 compounds was selected in the case of the OtavaSH2 data set, 20 in the case of the NP data set, 20 in the case of the Benchmark data set, 20 in the case of the Mcule-in-stock data set, and 10 in the case of the Enamine REAL data set, with half and half of the compounds being selected based on docking score (docking score ≤ −5.5) and DS/HA values (DS/HAN ≤ −0.2), respectively. We note here that the number of received and measured compounds might differ from these numbers due to unavailability at the moment of purchasing, poor solubility, or unsuccessful synthesis in the case of the Enamine REAL data set.

In general, small molecules were selected by visually inspecting their binding pose, while placing great emphasis on chemical diversity. Specifically, several structural and binding mode aspects were defined as guidance for compounds to be considered for purchasing and measurement. In terms of binding pose, a compound was eligible for being a virtual hit, if it interacted with the key anchoring residue defined in the appropriate H-bond constraint (R609 for STAT3 SH2 domain, R618 for STAT5b SH2 domain), AND it had at least a total of two advantageous interactions with the protein (in addition to the anchoring residue), AND did not have a significant part of it in a solvent-exposed region (no contact with the protein). Structurally, a compound was considered if it had more than ten but less than 50 heavy atoms, had less than four amide bonds, and had a maximum of two carboxylic groups.

To cover as large a chemical space as possible for prospective screening, if two compounds had very similar structures (e.g., only differing by a single heavy atom or a single functional group), only one was purchased, with the lower docking score value being favored. (The only exceptions here were compounds S5_M1 and S5_M7 that only differed by a single hydroxylic group but displayed excellent DS/HA values of −0.417 and −0.393, respectively, and were both purchased and tested).

#### Benchmarks and Evaluation

To benchmark the relevant docking score and DS/HA distributions, we have docked 20,000 (Mcule-in-stock) or 55,000 (Enamine REAL) randomly selected compounds from the data sets with Glide SP: these sets constituted the basis of comparing the docking score distributions (see Discussion).

Additionally, to provide a more general benchmark case against the whole Deep Docking workflow, we have used the RDKit^61^ Diversity Picker node^62^ in KNIME,^63^ to select 117,500 chemically diverse compounds from the Mcule-in-stock database (Benchmark data set), which is the exact same number that is docked in total during the Deep Docking workflow. Then, we docked these compounds with Glide SP against the STAT3 SH2 domain and selected 15 compounds for measurements, to have a clear baseline to evaluate the Deep Docking workflow against. (The docking and LigPrep parameters for the benchmark approaches were the same as described earlier.)

In addition, to evaluate the performance of each Deep Docking run, the benchmarking statistics per iteration were also evaluated, including recall, precision, and ROC-AUC values. Recall and precision are defined as follows:

Precision=True⁣Positives⁣(TP)True⁣Positives⁣(TP)+False⁣Positives⁣(FP)⁣Recall=True⁣Positives⁣(TP)TruePositives⁣(TP)+False⁣Negatives⁣(FN)

These benchmarking statistics were plotted in Supporting Information Figure S6.

### Fluorescence Polarization Assay

The respective protocols for the expression and purification of the STAT3 SH2 ^86^ and STAT5b SH2 ^87^ domains were published in our recent works. Fluorescence polarization assays (FP-assays) were performed on a Molecular Devices SpectraMax iD5Multimode Microplate Reader (San Jose, CA, USA) using Greiner black 384-well flat-bottom nonbinding microplates with 40 μL final well volumes. The fluorescent peptides (5-FAM-G­(pTyr)­LPQTV-NH_2_ and 5-FAM-G­(pTyr)­LVLDKW-NH_2_, purchased from GenScript Biotech Ltd., Piscataway, NJ, USA), as well as the proteins, were diluted with a buffer containing 50 mM NaCl, 10 mM HEPES (4-(2-hydroxyethyl)-1-piperazineethanesulfonic acid), 1 mM EDTA (ethylenediaminetetraacetic acid), 2 mM DTT (dithiothreitol), and 0.1% Triton X-100, pH 7.5. For STAT3 (127–770), the final concentration of the protein was 200 nM, and the fluorescent peptide (5-FAM-G­(pTyr)­LPQTV-NH_2_) was added at a final concentration of 5 nM; for STAT5b (136–703), the final concentration of the protein was 500 nM, and the fluorescent peptide (5-FAM-G­(pTyr)­LVLDKW-NH_2_) was added at a final concentration of 10 nM. The wells were treated with varying concentrations of inhibitors (1250 to 0.11 μM final concentrations). The final DMSO content was 2.5%. After mixing, the protein and inhibitors were incubated at room temperature for 30 min, then the fluorescent peptide was added, and the plate was incubated for another 20 min prior to the fluorescence readout (extinction wavelength: 475 nm, emission wavelength: 520 nm). The measurements were carried out in at least three biological replicates. Fluorescence polarization was calculated from the perpendicular and parallel fluorescence intensities and then plotted against concentration. The IC_50_ values were determined after fitting quadratic dose–response curves on the data points in GraphPad Prism 8.0.1. A compound was identified as active, if it had a mean IC_50_ value under 100 μM. All plotted curves and data points for the hit compounds are included in Section 2 of the Supporting Information.

### Isothermal Titration Calorimetry

The detailed protocol for the expression and purification of the STAT5b-NTD was described in our recent work.^88^ ITC measurements were carried out on a MicroCal PEAQ-ITC microcalorimeter (Malvern Instruments, Worcestershire, UK). All protein and ligand solutions were composed of 1X PBS (pH 7.4) buffer containing 8% (V/V) DMSO to avoid buffer mismatch. Protein solutions containing 25–26 μM protein were prepared in batches prior to measurements by diluting 676 μM stock solution (stored in 1X PBS (pH 7.4) buffer at −80 °C) warmed to room temperature with 1X PBS (pH 7.4) buffer solution and an appropriate amount of DMSO. Ligand solutions were prepared by first dissolving the ligands in DMSO to acquire a 50 mM stock solution, which was then diluted to 1 mM (in the case of S5N_M2 ligand) or 4 mM (all other ligands) concentration using 1X PBS (pH 7.4) buffer solution. Appropriate amounts of DMSO were also added to the solution containing 1 mM ligand to yield a 8% (V/V) DMSO content. The protein solution was loaded into the sample cell in a volume of 200 μL and was titrated at 25 °C with the ligand solution at a stirring speed of 1000 rpm and a reference heat rate of 10 μcal/s in a high feedback mode. Used initial delay was 120 s, and the injection program was composed of an initial first injection with 0.5 μL over 2 s, followed by 18 injections with 2 μL over 4 s. Injections were performed every 180 s.^89^ Blank measurements consisting of titrating the ligand into the buffer (1X PBS (pH 7.4) containing 8% (V/V) DMSO) were carried out to correct for the heat of dilution for all ligands using the appropriate injection program. Data were analyzed using the MicroCal PEAQ-ITC analysis software (version 1.22) by fitting a single-site binding curve. A compound was identified as active, if it had a mean K_d_ value under 100 μM. Binding curves of the identified active compounds are included in Supporting Information Figures S3–S5.

## Results

The active form of the STAT proteins is the phosphorylated dimer form.^36,90,91^ The direct method to inhibit STAT protein dimerization includes direct small molecule binding to the SH2 domain^92^ or, as a new approach, the NTD.^93,94^ Out of the two target domains, the SH2 domain is the most often targeted, with the NTD being a novel target for inhibiting STAT activation.^88^ Numerous potent small molecules have been already published for the SH2 domain of STAT3 ^92,95–99^ and, to a lesser extent, STAT5b;^77–81,100^ however, by this date, there is still no clinically approved, direct STAT inhibitor.

Leveraging the available structural and biochemical information against the various STAT3 and STAT5b protein domains, we have utilized the thoroughly studied STAT3 SH2 domain as a primary benchmark of the DeepDocking workflow vs “traditional” (or “knowledge-based”) workflows, one of which applies “brute-force” docking of smaller libraries, the other using a diverse selection from a larger data set to produce a smaller, chemically diverse data set. Learning from our experiences here, we moved on to discover new inhibitors of more challenging PPI oncotargets: the less thoroughly explored STAT5b-SH2 and the completely novel STAT5b-NTD.

### Benchmarking and Method Comparison against the STAT3 SH2 Domain

#### ‘Knowledge-Based’ Approach

Virtual screening of the OtavaSH2 data set resulted in 27 compounds, virtual screening of the NP data set resulted in 17 compounds, and last, virtual screening of the Benchmark resulted in 15 compounds to be measured by the FP assay against the STAT3 SH2 domain. For the structures of all chosen compounds, as well as their docking scores, DS/HA, and IC_50_ values, please refer to the Supplementary Data. The FP-assay measurements identified one active compound from the OtavaSH2 data set, **S3_O10** (Figure 2), corresponding to a hit rate of 3.7% (1/27), while no active compound was identified from either the NP data set or the Benchmark data set. The hit compound contains a carboxylic group which interacts with the R609 residue, and it also contains a coumarin motif. A weakly binding compound (mean IC_50_ value higher than 100 μM) from the Benchmark data set (**S3_B12**) was also identified (included in the Supplementary Data).

**Figure 2:**
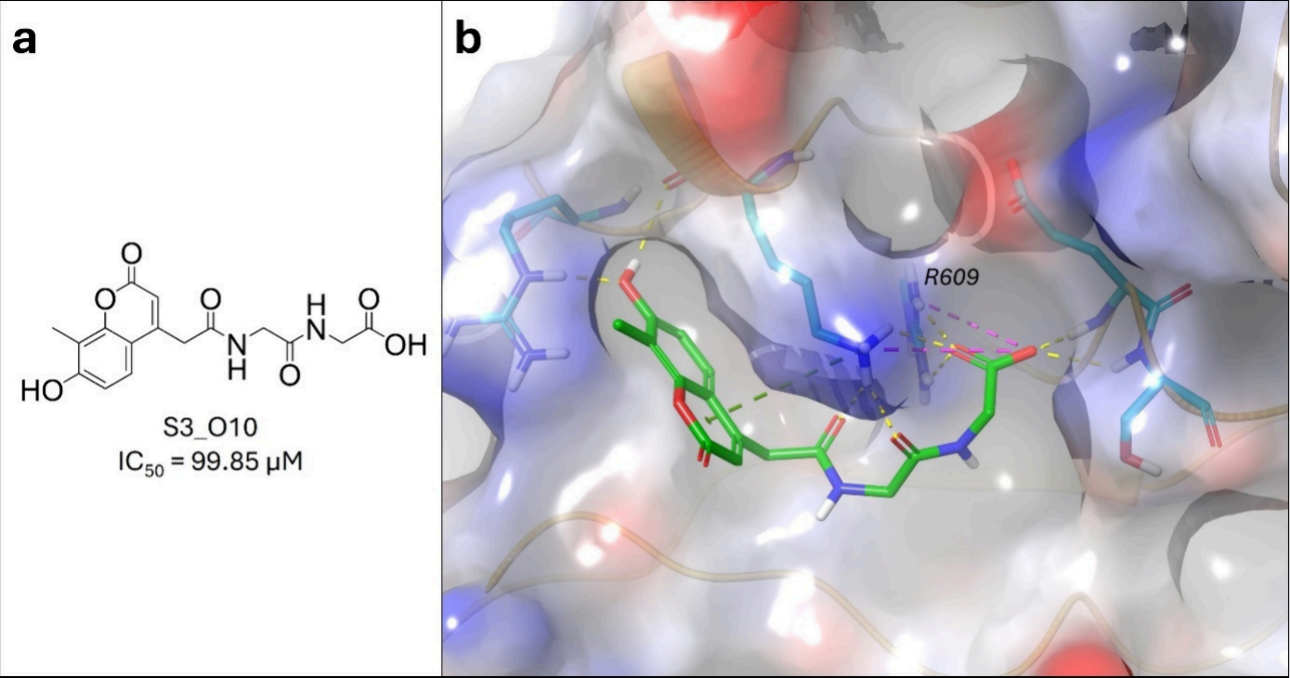
(a) Structures of the identified active from the OtavaSH2 data set against the STAT3 SH2 domain, with its corresponding mean IC_50_ value. (b) Binding mode of **S3_O10** against the SH2 domain of STAT3 (PDB ID: 6QHD).^69^

#### AI-Based Approach

The Deep Docking-assisted virtual screening of the Mcule-in-stock and Enamine REAL data sets resulted in 16 and eight compounds, respectively, to be ordered for FP-assay measurements against the STAT3 SH2 domain. For the structures of all chosen compounds, as well as their docking scores, DS/HA, and IC_50_ values please refer to the Supplementary Data. The FP-assay measurements identified three active compounds, **S3_M2**, **S3_M5**, and **S3_M13** from the Mcule-in-stock data set, and four active compounds, **S3_E4**, **S3_E5**, **S3_E6**, and **S3_E7** from the Enamine REAL data set (Figure 3), corresponding to hit rates of 19% (3/16) and 50% (4/8), respectively. One weakly binding compound from the Mcule-in-stock data set (**S3_M15**) and two weakly binding compounds from the Enamine REAL data set (**S3_E3** and **S3_E8**) were also identified (included in Supplementary Data).

**Figure 3:**
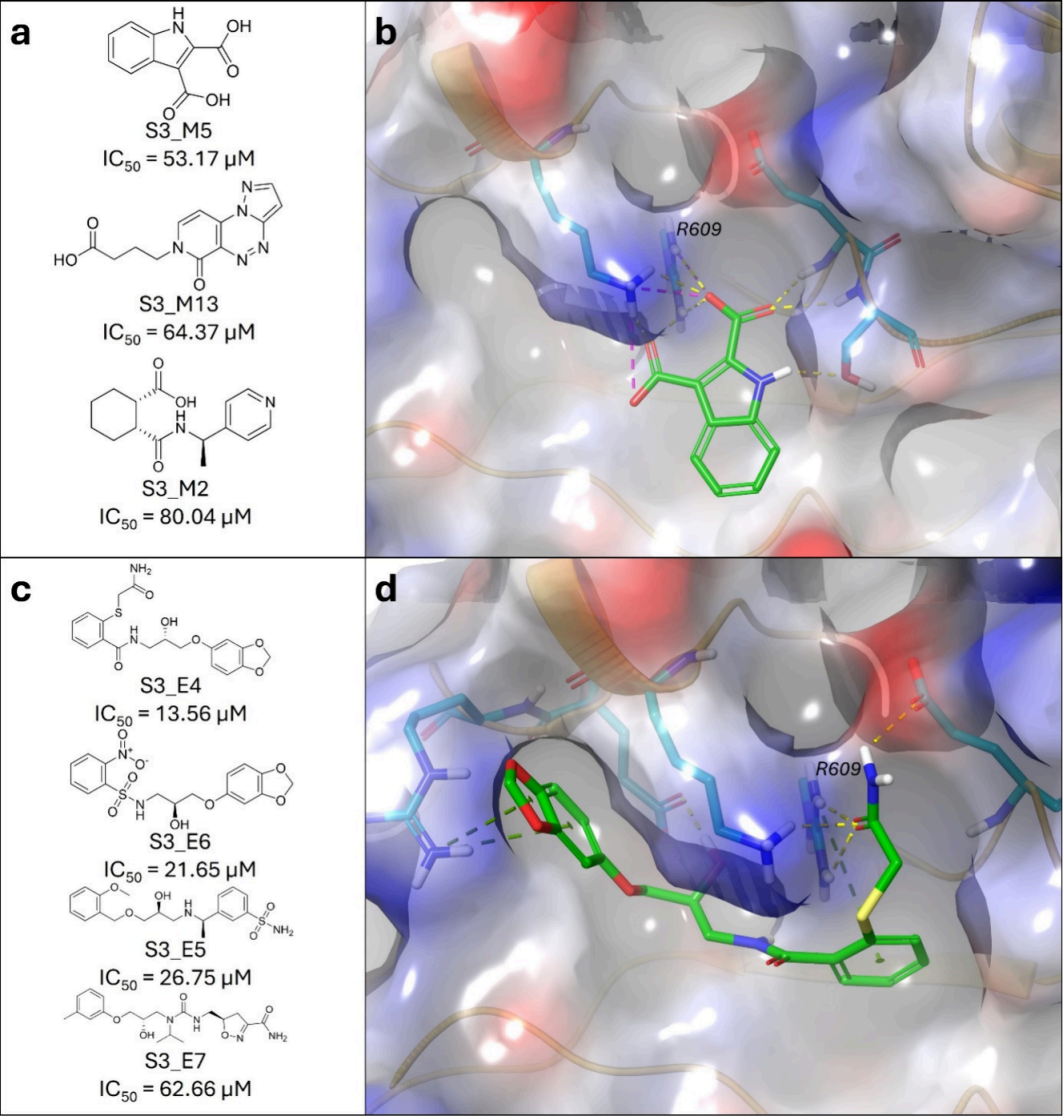
(a) Structures of the identified actives from the Mcule-in-stock data set against STAT3 SH2 domain, with their corresponding mean IC_50_ values. (b) Binding mode of the most potent active compound from the Mcule-in-stock data set, **S3_M5**, against the SH2 domain of STAT3 (PDB ID: 6QHD).^69^ (c) Structures of the identified actives from the Enamine REAL data set against STAT3 SH2 domain, with their corresponding IC_50_ values. (d) Binding mode of the most potent active compound from the Enamine REAL data set, **S3_E4**, against the SH2 domain of STAT3 (PDB ID: 6QHD).^69^

The identified active compounds from the Mcule-in-stock all contain carboxylic acid groups (one or two) which interact with the R609 residue, resulting in their stronger affinity toward STAT3.

The two most potent active compounds from the Enamine REAL, **S3_E4** and **S3_E6**, both have IC_50_ values in the low/mid micromolar range and both contain a 1,3-benzodioxole motif. These compounds have a very similar binding pose, with the 1,3-benzodioxole interacting with the R595 residue, which is another key residue for inhibition alongside R609, and should explain their higher affinity.^101^

We note here that the activities detected for the hit compounds described in this work are consistent with the IC_50_/K_i_ range of 0.52–114 μM for reported STAT3 inhibitors that were discovered by virtual screening.^17^ (Nonetheless, we have used the Aggregator Advisor tool^102^ to double-check the hits for possible artifacts: the script has not flagged any of the compounds as being similar to known aggregators.)

### The Economic Screening Workflow Identifies New STAT5b SH2 Domain Binders

The results from the AI-based approach for the STAT3 SH2 domain showed that the Deep Docking assisted virtual screening of the Mcule-in-stock data set can produce sufficiently good results. To further analyze the performance of Deep Docking with a data set containing only millions of compounds, 14 compounds from only the Mcule-in-stock data set were ordered for FP-assay measurements against the STAT5b SH2 domain. For the structures of all chosen compounds, as well as their docking scores, DS/HA, and IC_50_ values, please refer to the Supplementary Data. The FP-assay measurements identified six active compounds, **S5_M1**, **S5_M2**, **S5_M7**, **S5_M8**, **S5_M9**, and **S5_M13**, from the Mcule-in-stock data set (Figure 4), corresponding to a hit rate of 42.9% (6/14). Out of the six identified actives, **S5_M1** and **S5_M7** are structurally similar compounds differing in one hydroxylic group interacting with the protein backbone at the L643 residue, which might explain its higher activity. One weakly binding compound (**S5_M6**) was also identified (included in Supplementary Data).

**Figure 4:**
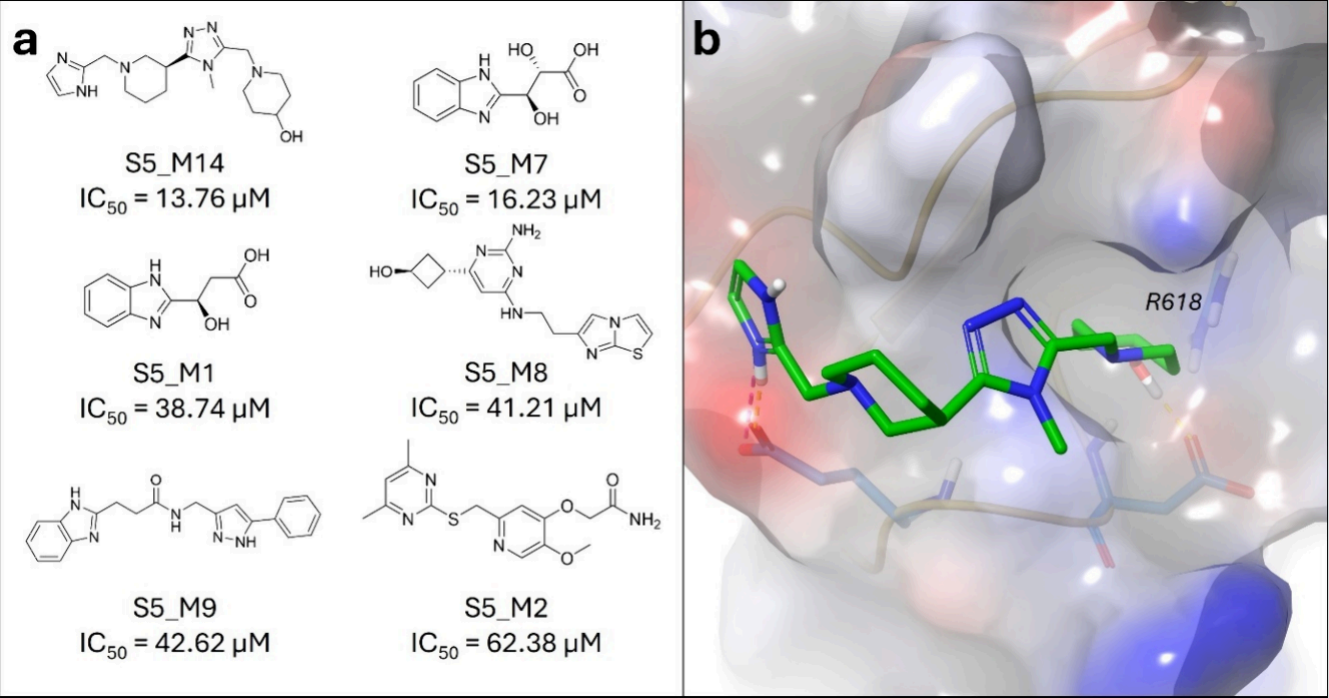
(a) Structures of the identified actives from the Mcule-in-stock data set against STAT5b SH2 domain, with their corresponding mean IC_50_ values. (b) Binding mode of the most potent active compound from the Mcule-in-stock data set, **S5_M14**, against the SH2 domain of STAT5b (PDB ID: 6MBW).^76^

#### Compound Activity Analysis

Just as for STAT3, we compared the activity of newly identified inhibitors against documented ones in the case of STAT5b. The ChEMBL database contains significantly less inhibitors against STAT5b with less than 700 Da molecular weight nd a reported IC_50_ value (15 compounds) than against STAT3 (235 compounds). Further analysis shows that, out of those 15 compounds, 12 compounds are from one series of analogs that went through chemical optimization with mean IC_50_ values ranging from 154 to 1400 nM,^103^ while the remaining three are from another series of analogs with mean IC_50_ values ranging from 1.4 to 37 μM.^104^ In our earlier work, we also collected STAT5b inhibitors identified via virtual screening, with IC_50_ or K_i_ values ranging from 9 nM to 80 μM.^17^

From the available data, we can conclude that the activity of the identified inhibitors in the current work fits into the activity range of already identified inhibitors, although they are on the weaker side. As described earlier in the case of STAT3, our main goal was to identify structurally diverse chemical starting points, and for that goal, these newly identified compounds are suitable for further chemical optimization against STAT5b. The small number of documented inhibitors for this target also makes any newly identified actives valuable.

To check if any of the identified compounds tend to aggregate, Aggregator Advisor was used.^102^ The script resulted in no measured compounds being similar to known aggregators.

### Prospective Use of the Economic AI-Based Workflow Identifies Dimerization Inhibitors of the Novel Oncotarget STAT5b N-Terminal Domain

Based on the excellent performance of the economic Deep Docking workflow in the two previous scenarios, we have applied this approach once again to discover dimerization inhibitors of the recently described oncotarget, the N-terminal domain of STAT5b. The Deep Docking-assisted virtual screening of the Mcule-in-stock data set resulted in 14 compounds to be ordered for ITC (isothermal titration calorimetry) measurements against the STAT5b NTD. For the structures of all chosen compounds, as well as their docking scores, DS/HA, and K_d_ values, please refer to the Supplementary Data. The ITC measurements identified three active compounds, **S5N_M2**, **S5N_M4**, and **S5N_M6** from the Mcule-in-stock data set (Figure 5), corresponding to a hit rate of 21.4% (3/14). Two weakly binding (mean K_d_ is higher than 100 μM) compounds (**S5N_M1** and **S5N_M7**) were also identified (included in Supplementary Data).

**Figure 5:**
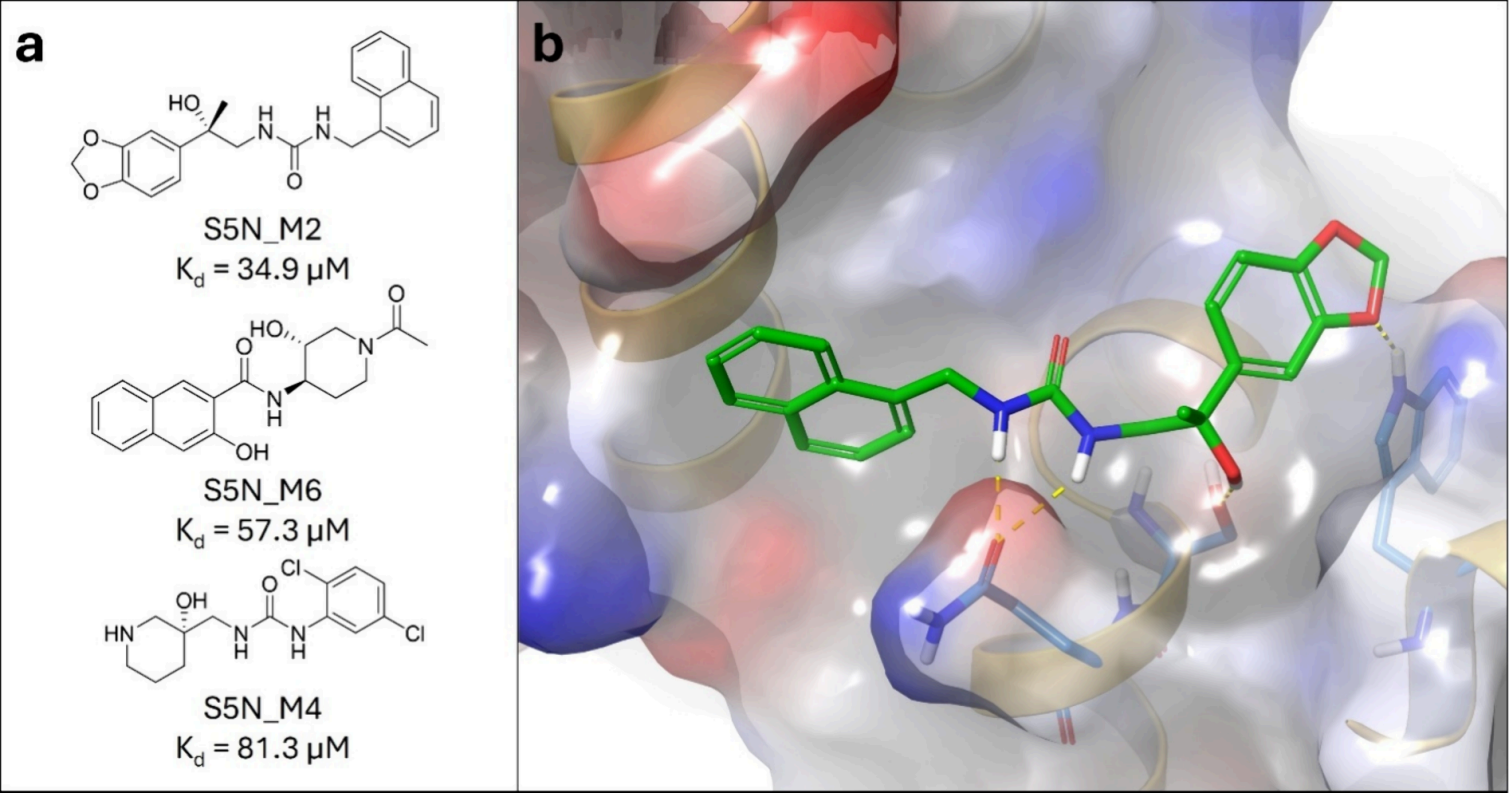
(a) Structures of the identified actives from the Mcule-in-stock data set against STAT5 NTD, with their corresponding mean K_d_ values. (b) Binding mode of the most potent active compound from the Mcule-in-stock data set, **S5N_M2**, against the NTD of STAT5b.

## Discussion

### Docking Score Distribution and Runtime Analysis

Computational resources used and hit rates for each virtual screening run are summarized in Table 3 (and Supporting Information Table S1 for the Benchmark data set). To compare the computational time requirement of each virtual screening run, the total CPU time in seconds was divided by the number of compounds within the data set (*t*_*one*_) (Table 3). We note here that for the Deep Docking runs, the time of the model training (which used GPUs) was included in the real time, but to compare the efficiency of the different workflows, the real time was multiplied with the number of used CPU cores to calculate the total CPU time and, from that, the *t*_*one*_ value. As such, the *t*_*one*_ value is a proxy that corresponds to an interpolated average time that would be required to process one compound if the whole data set was to be evaluated in the same way (vs. in reality, only a small fraction is docked in the Deep Docking workflow, while the rest are processed only by the deep learning model). For the virtual screenings in the ‘knowledge-based’ approach, the total CPU time (or likewise, the total elapsed time) was equal to the time required to complete the docking jobs, while in the AI-based approach, it is the time required to complete all 11 iteration steps (DL model training/refinement based on docking some compounds, and prediction of the rest of the data set) plus the time requirement to complete the post-DL docking jobs for the 20,000 (Mcule-in-stock) or 55,000 (Enamine REAL) predicted virtual hits. For virtual screenings done using Deep Docking, the CPU time was divided with the total number of compounds within the data set, instead of the number of actually docked compounds to reflect the much greater time efficiency.

**Table 3:** Computational Resource Requirements and Hit Rates Summarized for Each Virtual Screening Run

Target	STAT3 SH2 domain				STAT5b SH2 domain	STAT5b NTD
Data set	OtavaSH2	NP	Mcule-in-stock	Enamine REAL	Mcule-in-stock	Mcule-in-stock
Used approach	Knowledge-based	Knowledge-based	AI-based	AI-based	AI-based	AI-based
No. compounds in the data set	1,807	193,757	5,591,127	5,509,669,531	5,591,127	5,591,127
No. actually docked compounds^a^	1,807	193,757	117,500	13,055,000	117,500	312,500
Used CPU	Intel Xeon CPU E5–1660 v3	Intel Xeon CPU E5–1660 v3	AMD EPYC 7302P	HPC (AMD EPYC 7763)	AMD EPYC 7302P	AMD EPYC 7302P
No. CPU cores used	2	4	12	64	12	12
Used GPU	-	-	NVIDIA GeForce RTX 4070	HPC (NVIDIA A100)	NVIDIA GeForce RTX 4070	NVIDIA GeForce RTX 4070
No. GPUs used	0	0	1	12	1	4
Model training real time (s)	-	-	855,989	686,357	751,727	280,664
Total real time (s)	14,313	807,653	1,830,125	1,997,656	1,254,372	732,463
CPU time (CPU s)	28,626	3,230,612	21,961,500	127,849,984	15,052,464	8,789,556
Calculated time requirement for one compound with one CPU core (*t*_*one*_) (s)^b^	15.841	16.674	3.9279	0.023205	2.6922	1.5721
No. compounds purchased and measured	27	17	16	8	14	14
No. hits	1	0	3	4	6	3
Hit rate (%)	3.7	0.0	18.8	50.0	42.9	21.4

a For the OtavaSH2 and NP data sets, this number is equal to the number of compounds in the data set, while for the Mcule-in-stock and Enamine REAL data set, this number is equal to the total number of compounds docked in each Deep Docking iteration plus the number of compounds docked after the Deep Docking iterations (20,000 for Mcule-in-stock, 55,000 for Enamine REAL).

b CPU time divided by the size of the data set.

Based on the *t*_*one*_ values, AI-based virtual screenings were around one magnitude faster for the Mcule-in-stock data set and around three magnitudes faster for the Enamine REAL data set, as compared with the traditional VS approach. It is important to note here that Deep Docking consists of multiple steps (model training, model evaluation, virtual hit prediction) alongside docking, whose efficiency is mainly affected by the number of CPUs used. By contrast, correct parallelization, resource management, and optimization have a greater importance for efficient AI-based virtual screening, compared to a traditional docking calculation. The virtual screenings using the Mcule-in-stock data set were only 6.80 times slower against the STAT3 SH2 domain than using the NP data set, while covering 28.9 times more compounds, showcasing the efficiency of the Deep Docking workflow. By comparison, moving from the million-sized Mcule-in-stock set to the billion-sized Enamine set (3 orders of magnitude) conveys only a 6× increase in CPU time, highlighting the added value of the all-around use of GPUs, both for docking and model training. There are more peculiar data pairs in Table 3 as well: the virtual screening run against STAT5b NTD took almost half as much CPU time to complete as the one against the STAT5b SH2 domain on the same infrastructure, while docking 2.66 times more compounds. Reasons may include the larger size of the docking grid and the use of an H-bond constraint for the SH2 domain as well as the dependence of the speed of docking upon molecular complexity, combined with the sampling characteristics of the Deep Docking workflow. That is, if a fairly complex molecule (whose docking is relatively slow) achieves a good docking score, Deep Docking labels it as a hit, and further iterations may include similarly large molecules, increasing the overall time required for the docking phases.

Comparing the hit rates (Table 3), virtual screenings with the OtavaSH2 data set (3.7%) against the STAT3 SH2 domain and with the Mcule-in-stock data set against the STAT3 SH2 domain (18.8%) and STAT5b-NTD (21.4%) resulted in hit rates between 1% to 40%, which is the typical hit rate range in virtual screening.^105^ By comparison, screening the Benchmark data set against the STAT3 SH2 domain resulted in only a single weakly binding compound (S3_B12, with a mean IC_50_ value slightly higher than 100 μM) from the 15 measured compounds (technically a 0% hit rate), highlighting the added value of Deep Docking in hit identification. In the case of virtual screening with the Enamine REAL data set against the STAT3 SH2 domain and the Mcule-in-stock data set against the STAT5b SH2 domain, hit rates were 50.0% and 42.9%, respectively, which are exceptionally good values.

These results show that virtual screening with an AI-based approach can cover significantly more compounds within the same amount of time, while also being capable of producing better or, in some cases, exceptionally high hit rates. The results also show that an AI-based approach can work well even with evaluating a smaller data set.

Docking score distributions were analyzed in Figure 6. For the ‘knowledge-based’ approach, all compounds from the OtavaSH2 and NP data sets were evaluated, while for the AI-based approach, the exported top 20,000 compounds from the Mcule-in-stock data set and the exported top 55,000 compounds from the Enamine REAL data set by Deep Docking were evaluated. For additional information about the effectiveness of Deep Docking to enrich compounds with low (favorable) docking scores, a randomly selected set of 20,000 (Mcule-in-stock) or 55,000 compounds (Enamine REAL) was also evaluated. A lower docking score correlates with a predicted stronger affinity; however, larger molecules with more heavy atoms tend to have a bias toward low docking score values. To eliminate this bias, the docking score values were divided with the compounds’ heavy atom count (HA) to get DS/HA values.^106^ The distributions of the DS/HA values are also plotted in Figure 6.

**Figure 6:**
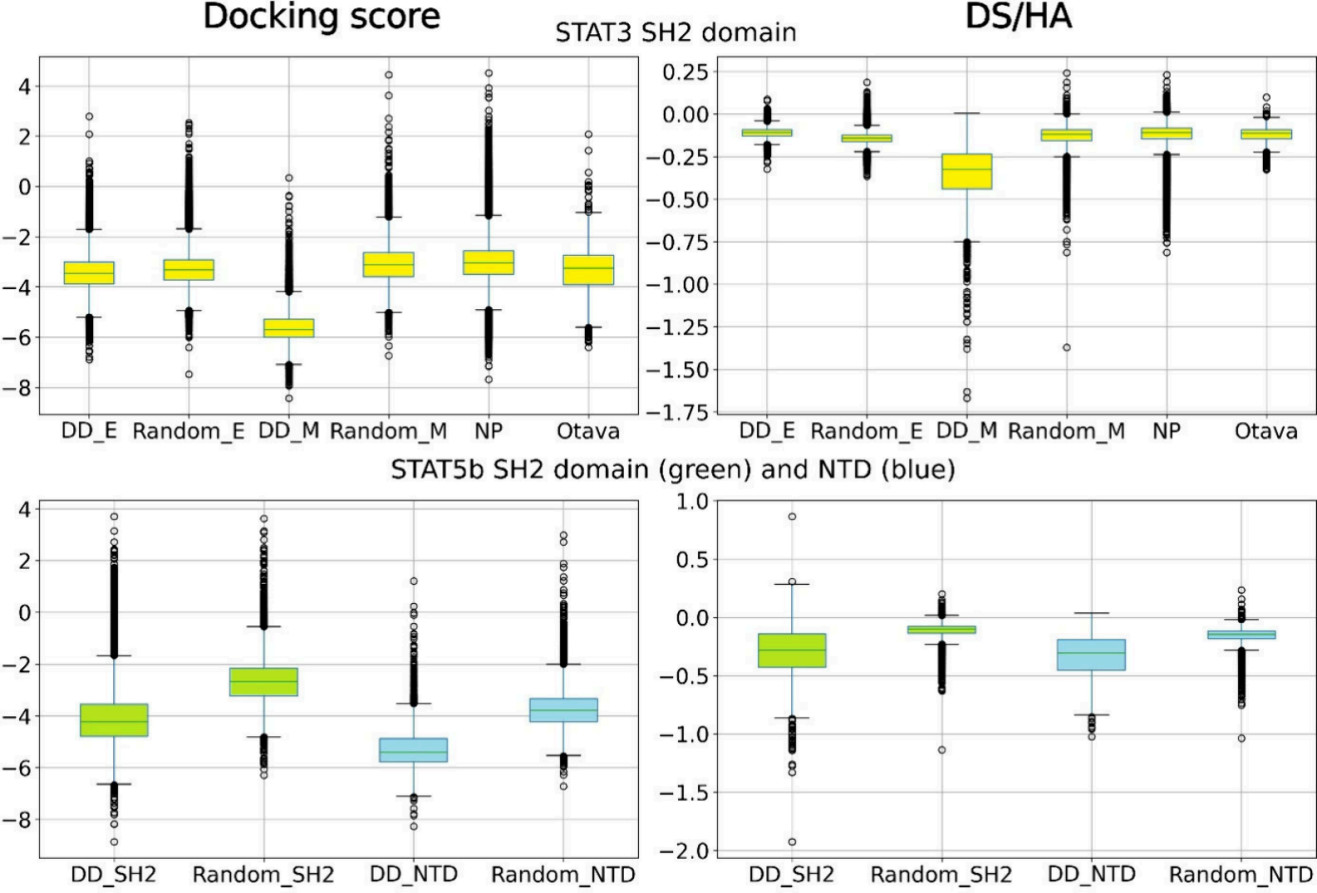
Docking score and DS/HA distributions of docked compounds in the box and whisker plots. In the case of the STAT3 SH2 domain, for the NP (NP column) and OtavaSH2 (Otava column) data sets, the distributions correspond to all compounds, while for the AI-based virtual screenings, they correspond to the docking scores and DS/HA values of the top 20,000 (DD_M column) or 55,000 (DD_E column) predicted virtual hits by Deep Docking from the Mcule-in-stock and Enamine REAL data sets, respectively. Random_M and Random_E columns correspond to the docking score and DS/HA distributions for 20,000 or 55,000 randomly selected compounds from the corresponding data set, respectively. In the case of the STAT5b SH2 domain (colored green) and STAT5b NTD (colored blue), the DD columns (DD_SH2 and DD_NTD) correspond to the docking score and DS/HA distributions for the top 20,000 compounds from the Mcule-in-stock data set for the appropriate target, while the Random columns (Random_SH2 and Random_NTD) correspond to the docking score and DS/HA distributions for the 20,000 randomly selected compounds from the Mcule-in-stock data set for the appropriate target.

For the virtual screens against the STAT3 SH2 domain, the docking score distributions were similar for the Enamine REAL, OtavaSH2, and NP data sets, while the Mcule-in-stock data set shows a clear shift toward lower docking score values. Comparing the results from the Deep Docking runs and the results from the randomly selected compounds, the run with the Mcule-in-stock data set shows a clear shift toward lower docking score values, meaning that Deep Docking successfully enriched the compounds with low docking score compounds. Interestingly, the Deep Docking run with the Enamine REAL data set only produced slightly improved results; however, in concordance with its main objective of identifying strong outliers, it produced numerous compounds with docking score values below −6, which the random selection did not. The insignificant shift of the overall distribution could be explained by the fact that only 0.24% of the whole data set was actually evaluated with docking, vs 2.1% in the case of the Mcule-in-stock set. Another possible explanation is the usage of AutoDockGPU as the docking algorithm instead of Glide SP, as its different pose prediction performance influenced the binding energy ranking and, ultimately, the model training.

Regarding the DS/HA values, just as for the docking score distributions, only the Deep Docking run with the Mcule-in-stock data set showed a clear shift toward more negative values, while also having numerous outliers in the more negative DS/HA value region. As the DS/HA value can be considered a computational proxy of size-independent ligand efficiency, its distribution also gives information about the similarity of compounds regarding the marginal benefit of adding more heavy atoms: a smaller range of data means a more homogeneous marginal benefit for growing the compounds. Here, all DS/HA distributions are fairly narrow, with the exception of the Mcule-in-stock data set sampled by the Deep Docking workflow. By contrast, there is little difference between the DD-prioritized portion of the Enamine REAL data set vs a random selection, which might be explained by the lower coverage of this data set by docking (see above) and/or the finite set of chemical transformations and building blocks composing the Enamine REAL space vs the less restricted, albeit much smaller, Mcule-in-stock set.

For the virtual screenings against the STAT5b SH2 domain and NTD, only the AI-based workflow was executed, but a comparison with a randomly drawn set of molecules reinforces the findings reported above. More precisely, the Deep Docking runs produced a larger amount of more negative outliers and a better all-around result than random selection.

These results, especially those on the Enamine REAL data set, show that even without a systematic shift toward a more favorable docking score distribution, the Deep Docking workflow can reliably identify strong outliers, translating into an increased chance of identifying active compounds, ultimately resulting in exceptional hit rates (42.9% for STAT5b SH2 domain and 50.0% for STAT3 SH2 domain). As for the ‘knowledge-based’ approaches, interestingly the virtual screen with the NP data set did not produce any actives, despite a comparatively high number of strong outliers (vs the OtavaSH2 set) and our hypothesis that the larger complexity of these compounds would be beneficial for targeting more challenging, shallow, and diffuse PPI sites.

In the case of the OtavaSH2 domain, which (after the NP set) produced the lowest hit rate (3.7%) from the largest set of compounds tested (27 for STAT3-SH2), there were not many strong outliers in either docking score or DS/HA values. (This ultimately corroborates the lower chances of hit identification expected from compounds having more positive docking score values.) The lack of hits from the NP data set also challenges the notion of selecting a screening deck based on simple heuristics (e.g., “more complex molecules will confer a higher probability for finding hits against difficult PPI binding sites”) and highlights the added value of screening a larger chemical space.

## Conclusions

In this work, we have evaluated the performance of the AI-based Deep Docking workflow to identify novel inhibitors against difficult PPI targets, against traditional or ‘knowledge-based’ workflows. The SH2 domain of the STAT3 and STAT5b proteins and the N-terminal domain of STAT5b, a novel oncotarget, were chosen as examples. For the ‘knowledge-based’ approach, we have used two data sets, one specifically designed for SH2 domains (OtavaSH2 data set) and the other containing natural products or natural product-like compounds (NP data set). For the AI-based approach, we have used two large, but different-sized data sets: the Mcule-in-stock set of 5.59 M commercially available compounds and the Enamine REAL set of 5.51B synthesizable, on-demand virtual compounds. We have also used a Benchmark data set created by diverse selection from the Mcule-in-stock data set.

Compared to a hit rate of 3.7% from the OtavaSH2 library and a surprising lack of hits from the NP set, the Deep Docking workflow resulted in exceptional hit rates of 18.8%, 42.9%, and 21.4% from the Mcule-in-stock library (against STAT3-SH2, STAT5b-SH2, and STAT5b-NTD, respectively) and a 50.0% hit rate from the Enamine REAL library against the STAT3 SH2 domain.

In terms of runtime, while the AI-based approach requires more total computational time in accordance with the larger number of compounds within the data set, the projected CPU time of evaluating a single compound is multiple orders of magnitude smaller. In addition, the results show that the AI-based approach can produce better hit rates even with using a relatively small number of compounds for model training (i.e., fewer than in its originally intended use) than does the ‘knowledge-based’ approach after docking an altogether comparable number of compounds. Practically, if a docking of ∼ 120,000 is required in a ‘brute-force’ way (docking every single compound), it is highlighted here as a better alternative to run an AI-based virtual screening with a data set containing 5–10 million compounds instead, which is still feasible without supercomputing resources and is expected to produce better hit rates.

Docking score and DS/HA distributions were also analyzed, showcasing the superior ability of the AI-based approach to identify strong outliers with large negative docking scores (and thus increase the change of identifying hits), compared to both the ‘knowledge-based’ approach and a randomly selected set.

Lastly, we highlight that the reported virtual screens have successfully identified several new inhibitors of difficult PPI-type oncotargets. The activity ranges are in line with expectations of screening hits against this type of target, while the hit rates can be considered to be excellent or even exceptional for the AI-based workflow and in line with expectations for the traditional workflow (except for the NP set). While the STAT3 SH2 domain has a larger number of reported inhibitors, these compounds are a significant addition to the much smaller set of known STAT5b-SH2 inhibitors and, especially, to the reported inhibitors of the STAT5b N-terminal domain, where to the best of our knowledge, only two compounds were disclosed in our recent work.^88^ These results, together with the performance analysis of different virtual screening protocols, suggest utility of AI-based uHTVS workflows even on moderate infrastructure with a moderate-to-ultra large screening deck.

## Supplementary Material





## Data Availability

Training sets, docking and bioassay results and source data of the figures are published in the Supplementary Data file. Scripts used for the Deep Docking workflow are available at https://github.com/keserulab/uHTVS_toolkit.

## Notes

Structures (in SMILES format) of the used compounds in the training set for retrospective virtual screening against STAT3 SH2 domain and STAT5b SH2 domain; structures (in SMILES format) of the measured compounds from the virtual screening runs with their code names, IC_50_ or K_d_ values, docking scores and DS/HA values; docking scores and DS/HA values for all of the docked compounds; log­(IC_50_) values of the newly identified compounds vs the documented inhibitors from ChEMBL against STAT3 (XLSX)
Supplementary figures with the ROC curves from the results of the retrospective virtual screening against STAT3 SH2 domain (Figure S1) and STAT5b SH2 domain (Figure S2); FP-assay results for all hit compounds for STAT3 SH2 domain and STAT5b SH2 domain (pages S3–S7); ITC binding curves for all hit compounds for STAT5b NTD (Figures S3–S7); Table containing data about the virtual screening with the Benchmark data set (Table S1) Deep Docking benchmarking statistics for each Deep Docking run (Figure S8) (PDF)
