# Supplementary material for: Ultrahigh-Throughput Virtual Screening Strategies against PPI Targets: A Case Study of STAT Inhibitors
Source: J Chem Inf Model. 2025 Jul 4;65(14):7734–48. doi: 10.1021/acs.jcim.5c00907 (PMC12308805; doi:10.1021/acs.jcim.5c00907)
Supplement: Supplementary file 2 [file ci5c00907_si_002.pdf]

Ultrahigh-throughput virtual screening strategies against PPI targets: a case study of STAT inhibitors

Tibor Viktor Szalai,[1,2] Nikolett Péczka,[1,3] Levente Sipos-Szabó,[1,3] László Petri,[1] Dávid Bajusz,[1,*] György M. Keserű[1,3,*]

[1] *Medicinal Chemistry Research Group and Drug Innovation Centre, HUN-REN Research Centre for Natural Sciences, Magyar tudósok krt. 2, 1117 Budapest, Hungary*

[2] *Department of Inorganic and Analytical Chemistry, Faculty of Chemical Technology and Biotechnology, Budapest University of Technology and Economics, Műegyetem rkp. 3., H-1111 Budapest, Hungary*

[3] *Department of Organic Chemistry and Technology, Faculty of Chemical Technology and Biotechnology, Budapest University of Technology and Economics, Műegyetem rkp. 3., H-1111 Budapest, Hungary*

*Corresponding author emails: bajusz.david@ttk.hu, keseru.gyorgy@ttk.hu

Table of contents

1. ROC curves for the used protein structures....2

1.1. ROC curve for 6QHD (STAT3)....2

1.2. ROC curve for 6MBW (STAT5b)....3

2. Fluorescence polarization assay (FP-assay) results for the hit compounds....3

2.1. FP-assay results for STAT3 SH2 domain from the OtavaSH2 data set....3

2.2. FP-assay results for STAT3 SH2 domain from the Mcule-in-stock data set....4

2.3. FP-assay results for STAT3 SH2 domain from the Enamine REAL data set....5

2.4. FP-assay results for STAT5b SH2 domain from the Mcule-in-stock data set....5

2.5. FP-assay results for the weakly binding compounds....7

3. Isothermal titration calorimetry (ITC) results for the hit compounds....8

3.1. ITC results for STAT5b NTD from the Mcule-in-stock data set....8

3.2. ITC results for the weakly binding compounds....11

4. Runtime analysis for the Benchmark set....13

5. Deep Docking benchmarking statistics....14

1. ROC curves for the used protein structures

1.1. ROC curve for 6QHD (STAT3)

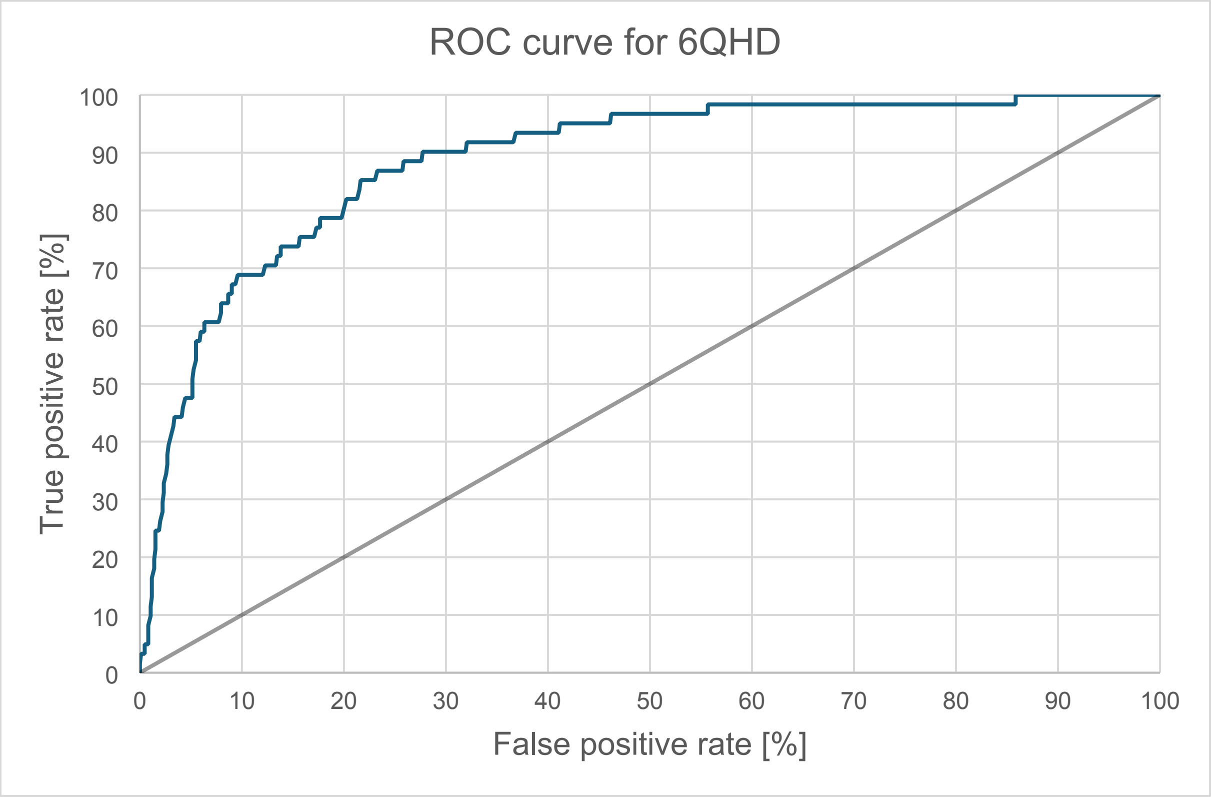

Figure S1. ROC curve for 6QHD.

1.2. ROC curve for 6MBW (STAT5b)

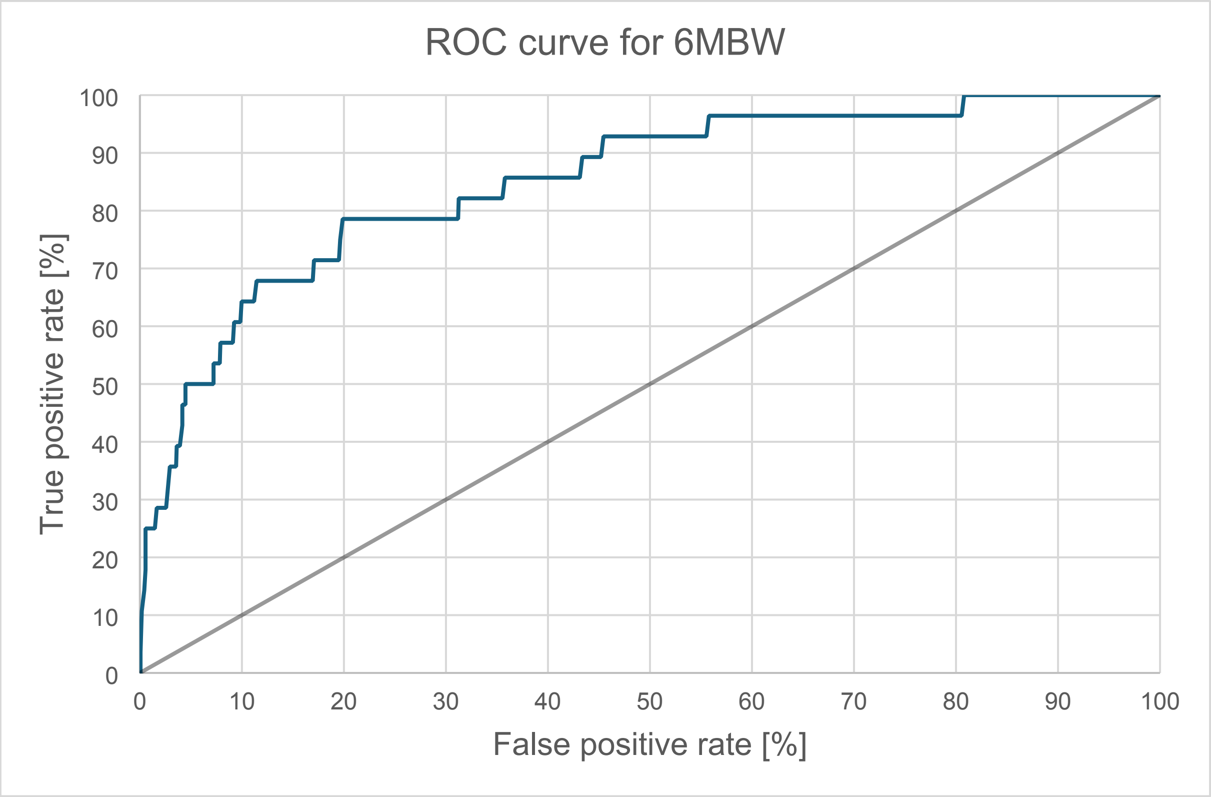

Figure S2. ROC curve for 6MBW.

2. Fluorescence polarization assay (FP-assay) results for the hit compounds

2.1. FP-assay results for STAT3 SH2 domain from the OtavaSH2 data set

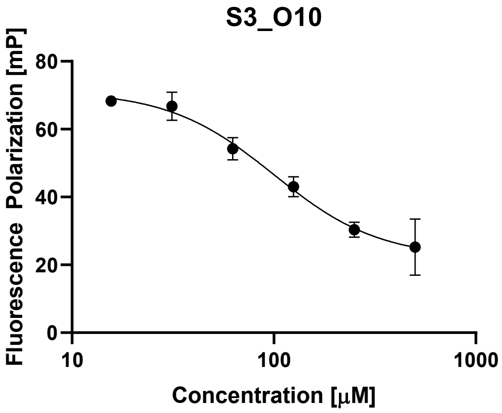

2.2. FP-assay results for STAT3 SH2 domain from the Mcule-in-stock data set

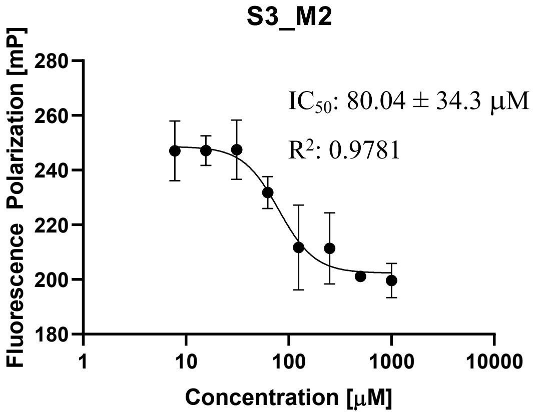

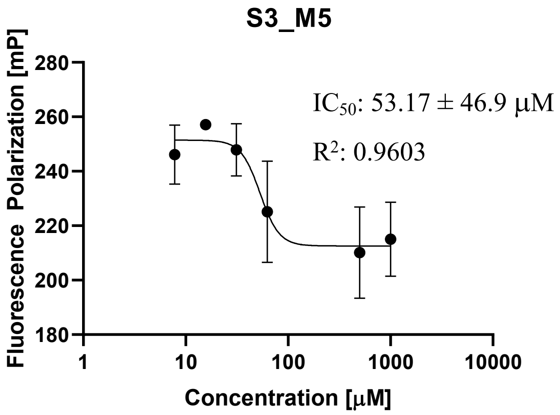

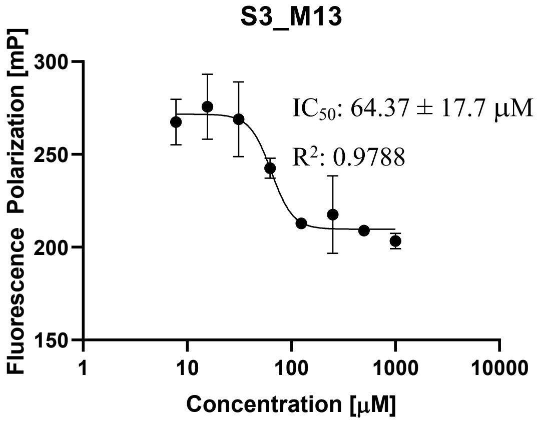

2.3. FP-assay results for STAT3 SH2 domain from the Enamine REAL data set

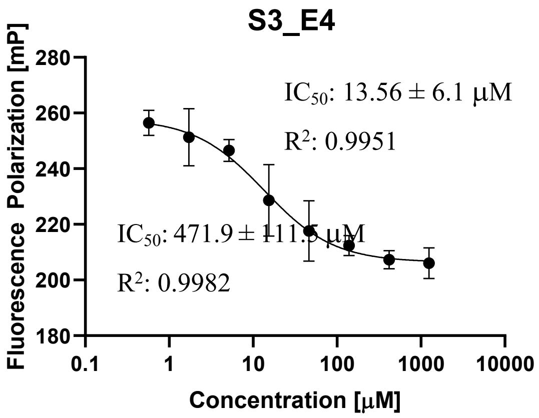

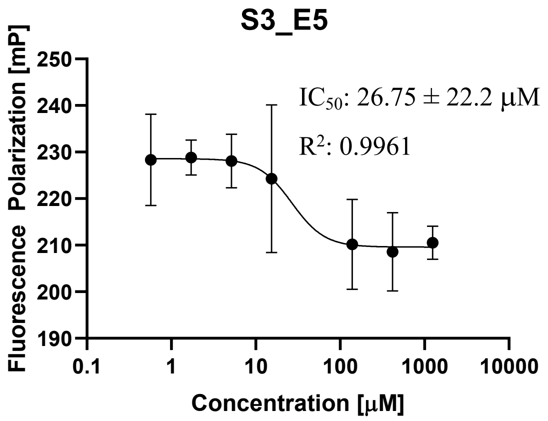

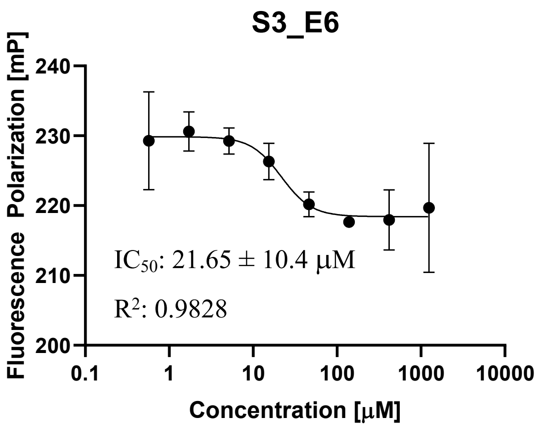

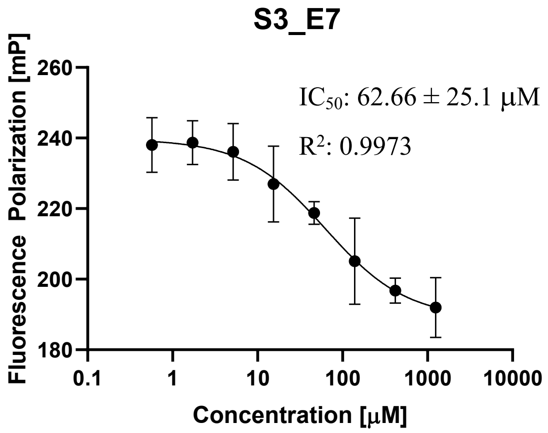

2.4. FP-assay results for STAT5b SH2 domain from the Mcule-in-stock data set

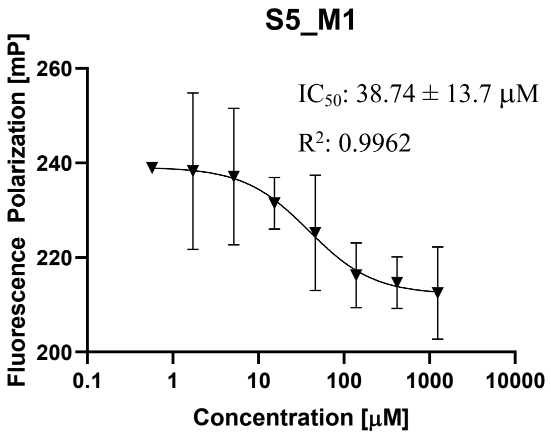

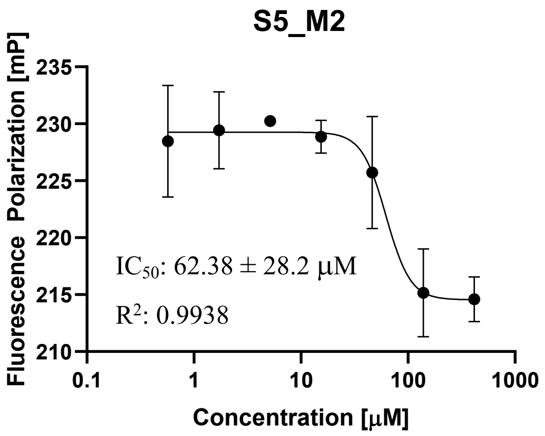

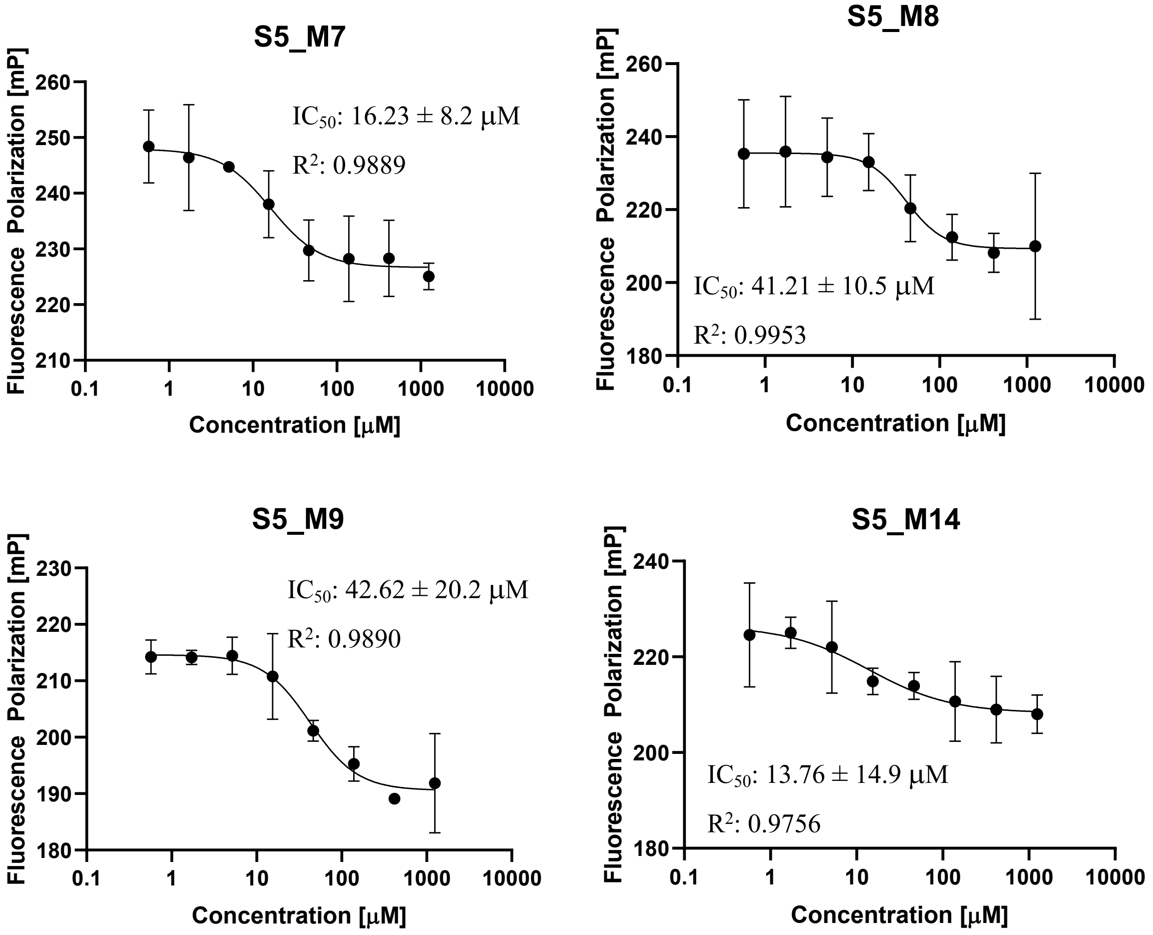
S5_M7
IC50: 16.23 ± 8.2 μM
R2: 0.9889
Fluorescence Polarization [mP]
Concentration [μM]
S5_M8
IC50: 41.21 ± 10.5 μM
R2: 0.9953
Fluorescence Polarization [mP]
Concentration [μM]
S5_M9
IC50: 42.62 ± 20.2 μM
R2: 0.9890
Fluorescence Polarization [mP]
Concentration [μM]
S5_M14
IC50: 13.76 ± 14.9 μM
R2: 0.9756
Fluorescence Polarization [mP]
Concentration [μM]

2.5. FP-assay results for the weakly binding compounds.

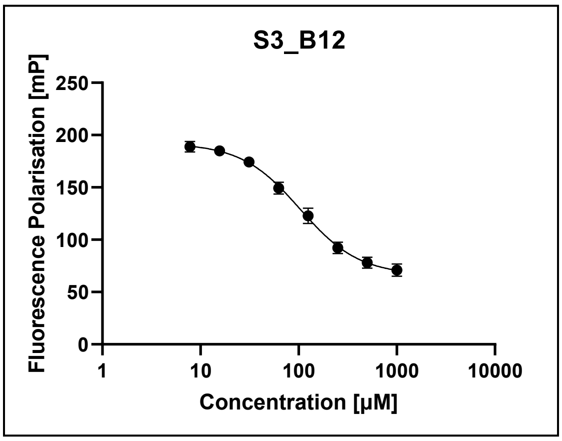

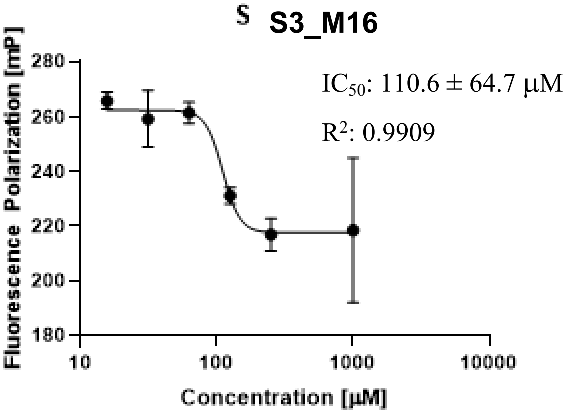

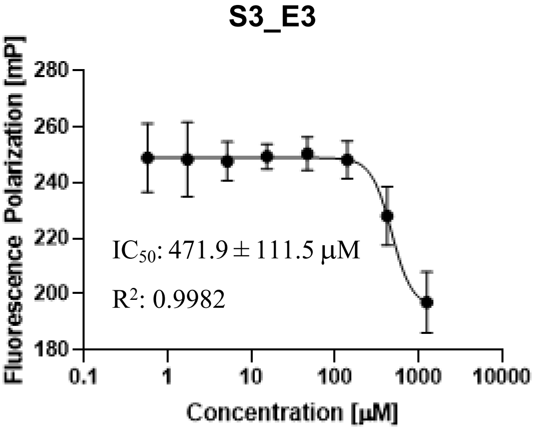

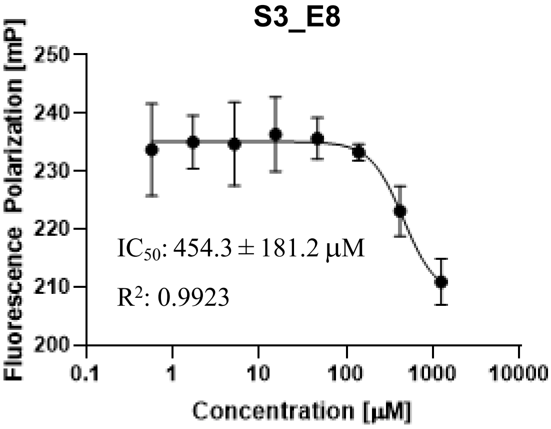

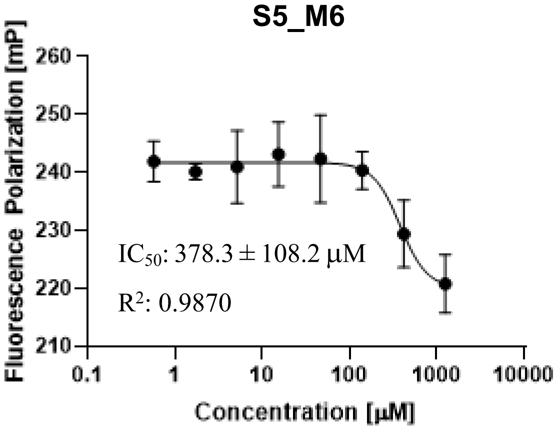

3. Isothermal titration calorimetry (ITC) results for the hit compounds

3.1. ITC results for STAT5b NTD from the Mcule-in-stock data set

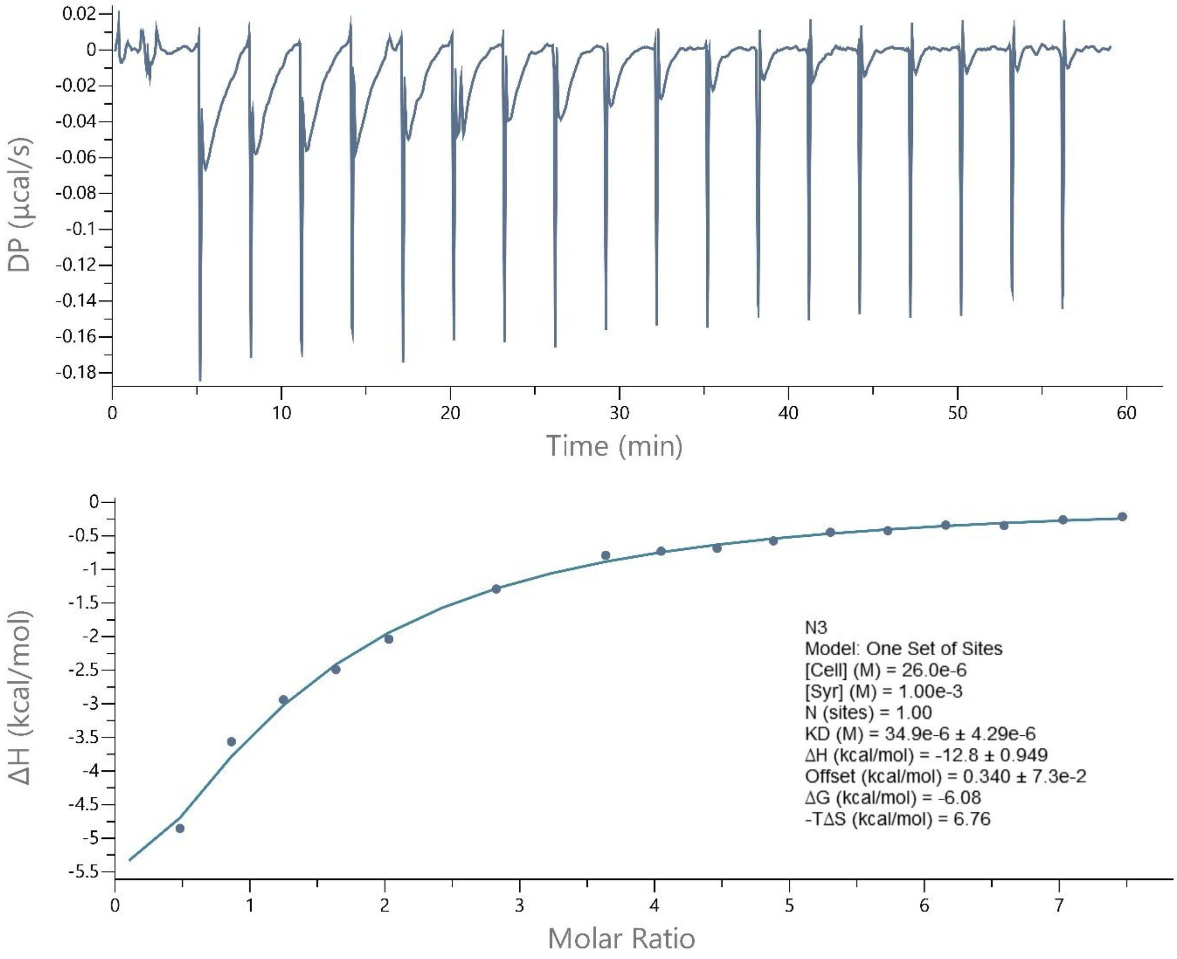

Figure S3. ITC results for S5N_M2. Blank measurement (ligand titrated to buffer solution) was subtracted from the data.

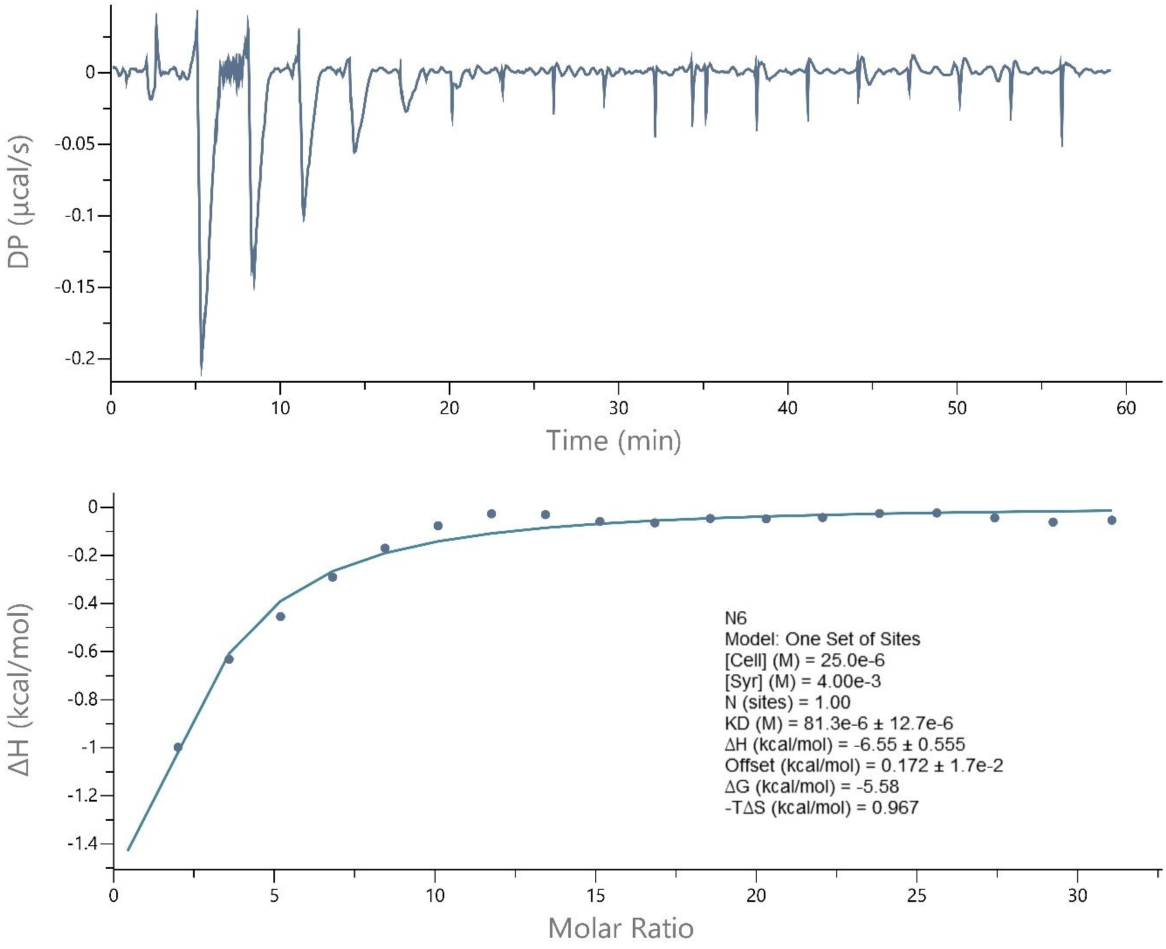

Figure S4. ITC results for S5N_M4. Blank measurement (ligand titrated to buffer solution) was subtracted from the data.

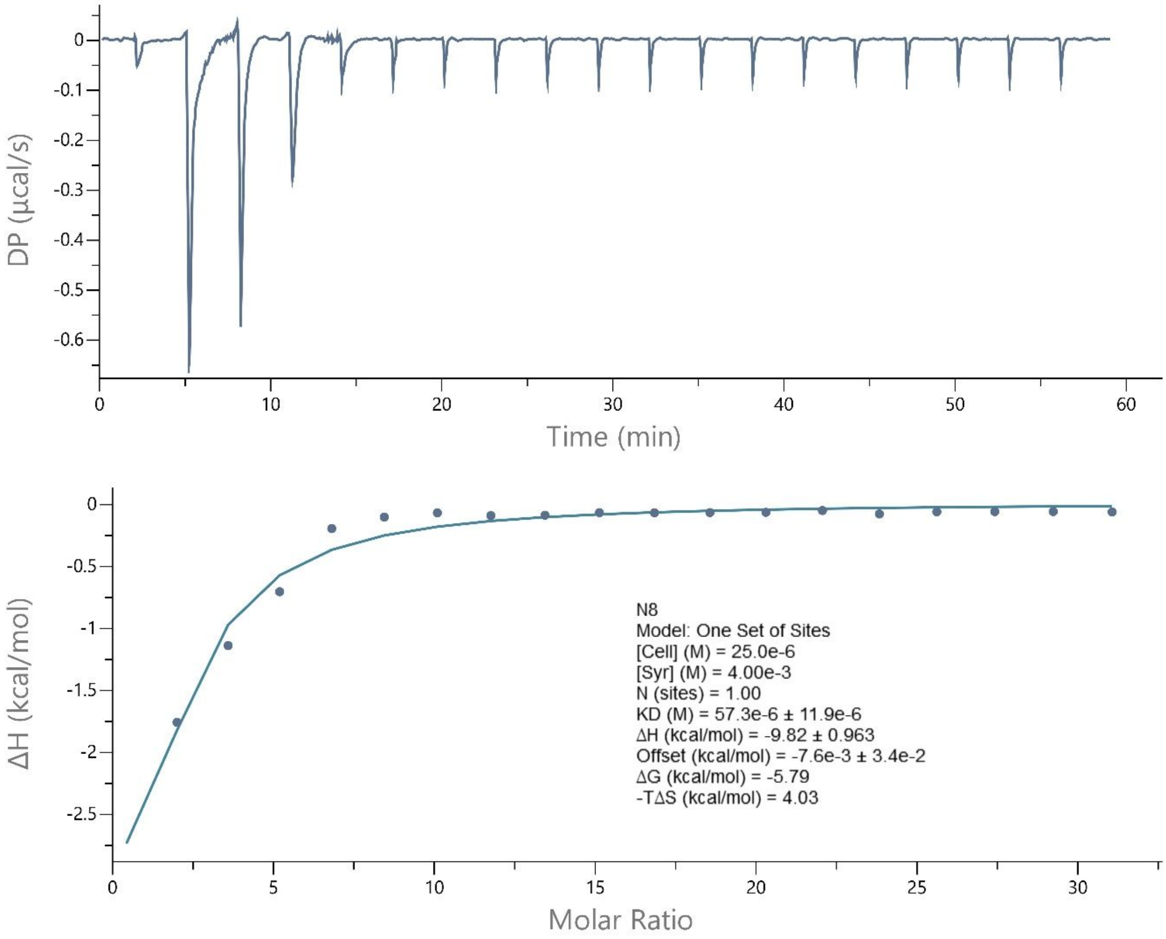

Figure S5. ITC results for S5N_M6. Blank measurement (ligand titrated to buffer solution) was subtracted from the data.

3.2. ITC results for the weakly binding compounds

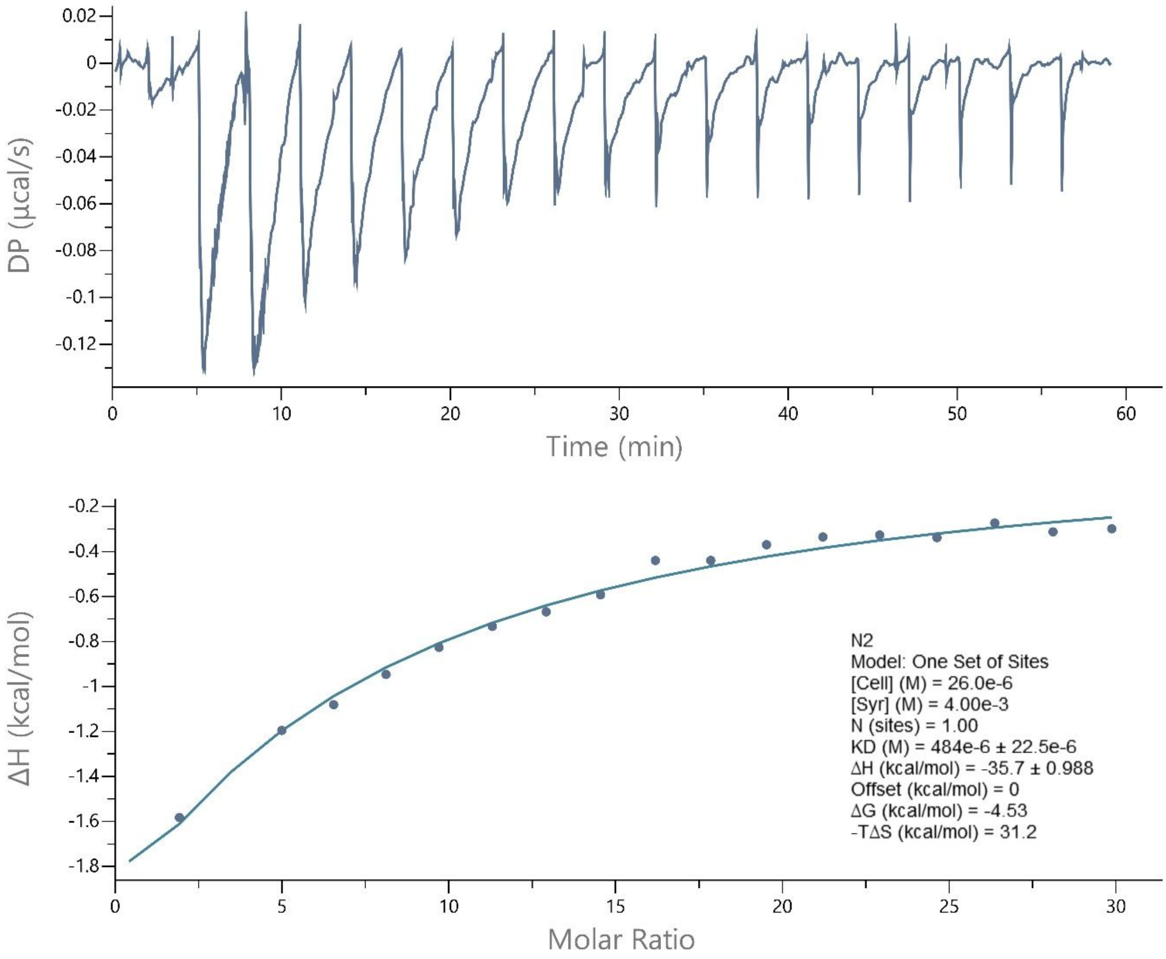

Figure S6. ITC results for S5N_M1. Blank measurement (ligand titrated to buffer solution) was subtracted from the data.

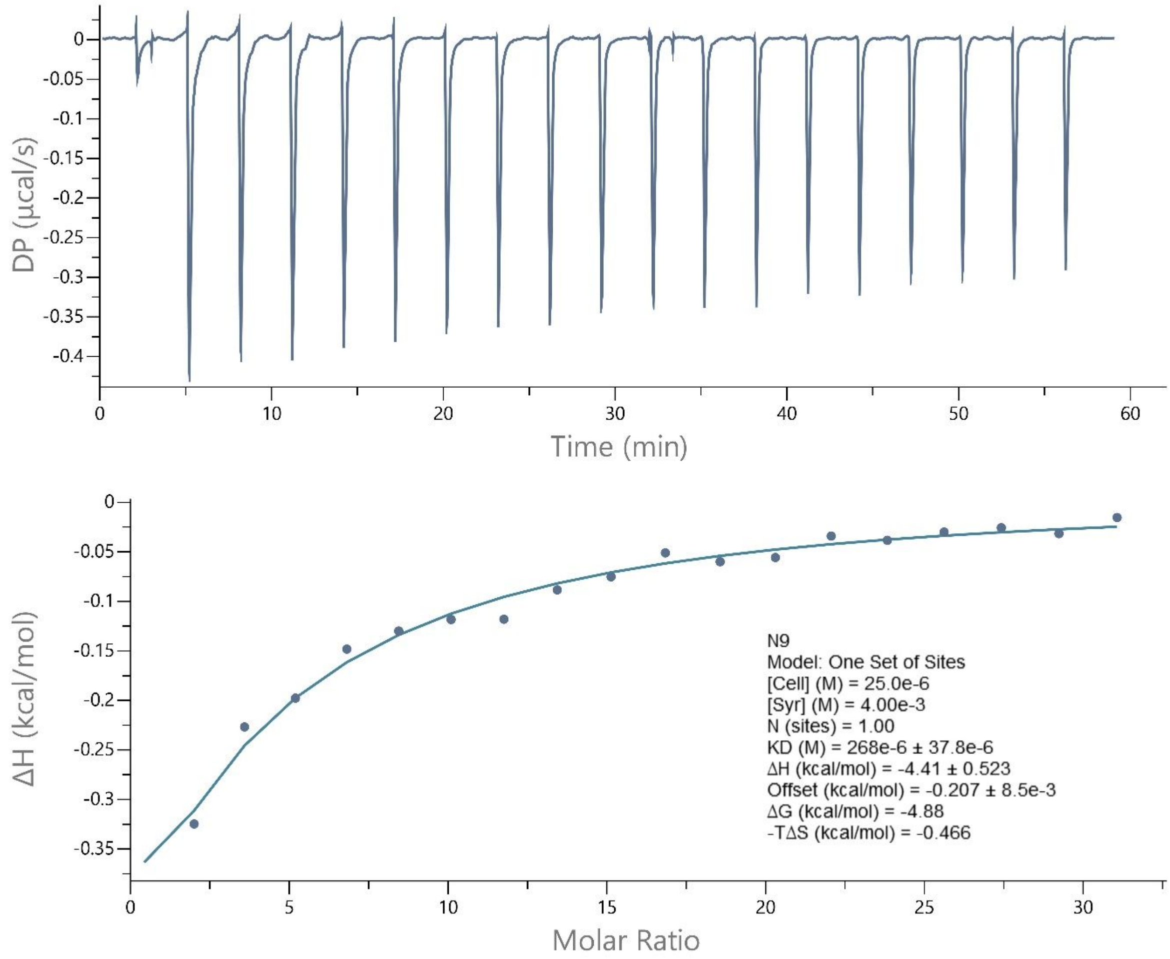

Figure S7. ITC results for S5N_M7. Blank measurement (ligand titrated to buffer solution) was subtracted from the data.

4. Runtime analysis for the Benchmark set

Table S1. Computational resource requirements and hit rates for the virtual screening runs with the Benchmark dataset and the Mcule-in-stock dataset with Deep Docking.

Target	STAT3 SH2 domain	
Dataset	Benchmark (derived from Mcule-in-stock)	Mcule-in-stock
Used approach	Knowledge-based	AI-based
No. compounds in the dataset	5,591,127	5,591,127
No. actually docked compounds	117,500	117,500
Used CPU	AMD EPYC 7451	AMD EPYC 7302P
No. CPU cores used	12	12
Used GPU	-	NVIDIA GeForce RTX 4070
No. GPUs used	0	1
Model training real time (s)	-	855,989
Total real time (s)	507,005	1,830,125
CPU time (CPU s)	6,084,060	21,961,500
No. compounds purchased and measured	15	16
No. hits	0	3
Hit rate (%)	0.0	18.8

5. Deep Docking benchmarking statistics

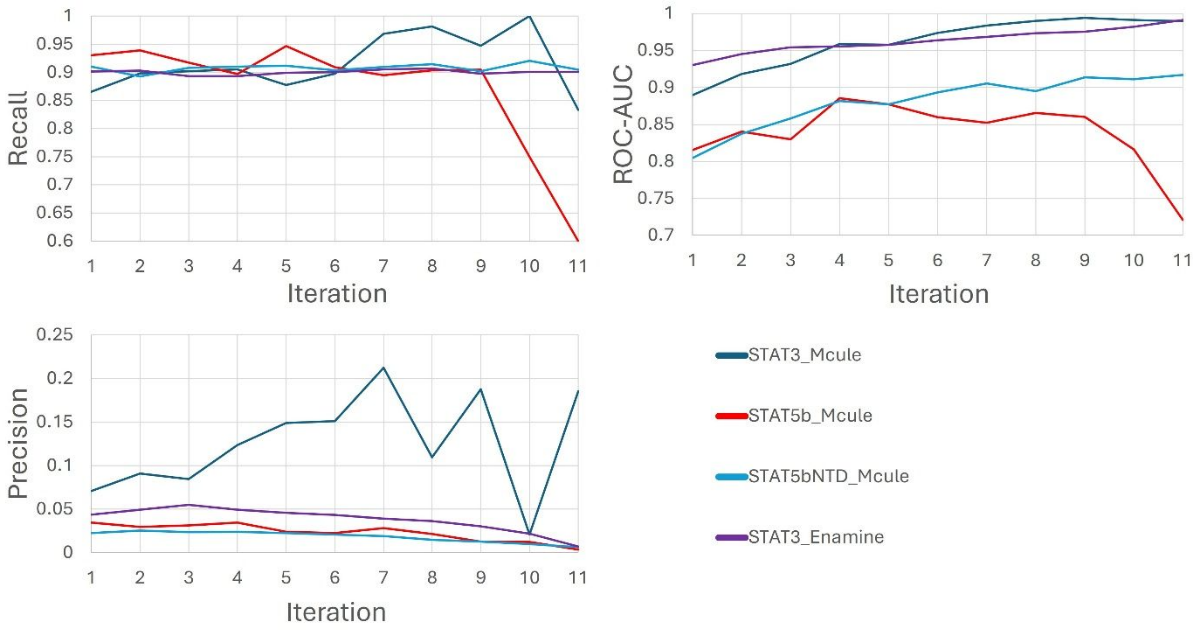

Figure S8. Benchmarking statistics (precision, recall, ROC-AUC) of each Deep Docking run.
